# Figures of merit and statistics for detecting faulty species identification with DNA barcodes: A case study in *Ramaria* and related fungal genera

**DOI:** 10.1371/journal.pone.0237507

**Published:** 2020-08-19

**Authors:** María P. Martín, Pablo P. Daniëls, David Erickson, John L. Spouge

**Affiliations:** 1 Department of Mycology, Real Jardín Botánico-CSIC, Madrid, Spain; 2 Department of Botany, Ecology and Plant Physiology, Campus Rabanales, University of Córdoba, Córdoba, Spain; 3 Joint Institute of Food Safety and Applied Nutrition, University of Maryland, College Park, Maryland, United States of America; 4 National Center for Biotechnology Information, National Library of Medicine, Bethesda, Maryland, United States of America; University of Helsinki, FINLAND

## Abstract

DNA barcoding can identify biological species and provides an important tool in diverse applications, such as conserving species and identifying pathogens, among many others. If combined with statistical tests, DNA barcoding can focus taxonomic scrutiny onto anomalous species identifications based on morphological features. Accordingly, we put nonparametric tests into a taxonomic context to answer questions about our sequence dataset of the formal fungal barcode, the nuclear ribosomal internal transcribed spacer (ITS). For example, does DNA barcoding concur with annotated species identifications significantly better if expert taxonomists produced the annotations? Does species assignment improve significantly if sequences are restricted to lengths greater than 500 bp? Both questions require a figure of merit to measure of the accuracy of species identification, typically provided by the probability of correct identification (PCI). Many articles on DNA barcoding use variants of PCI to measure the accuracy of species identification, but do not provide the variants with names, and the absence of explicit names hinders the recognition that the different variants are not comparable from study to study. We provide four variant PCIs with a name and show that for fixed data they follow systematic inequalities. Despite custom, therefore, their comparison is at a minimum problematic. Some popular PCI variants are particularly vulnerable to errors in species annotation, insensitive to improvements in a barcoding pipeline, and unable to predict identification accuracy as a database grows, making them unsuitable for many purposes. Generally, the Fractional PCI has the best properties as a figure of merit for species identification. The fungal genus *Ramaria* provides unusual taxonomic difficulties. As a case study, it shows that a good taxonomic background can be combined with the pertinent summary statistics of molecular results to improve the identification of doubtful samples, linking both disciplines synergistically.

## Introduction

Increasingly, scientists use DNA barcoding to identify the species of biological specimens. First, they extract short, standardized DNA sequences from the specimen [1]. Next, they compare the sequences to databases containing orthologous sequences from known species [2], with close matches indicating the taxonomy of the unidentified specimen. DNA barcoding has become important in many applications relating to the sustainability of natural resources, such as monitoring water quality (e.g. [3]), protecting endangered species (e.g. [4]), controlling agricultural pests (e.g. [5]), and identifying diseases (e.g. [6]), among many others (e.g., www.boldsystems.org).

Implicitly, any study estimating the accuracy of species identification by DNA barcoding relies on the choice of a taxonomic species concept [7, 8]. Traditionally, morphological features resolve the boundaries between neighboring species. If expert taxonomists disagree on species boundaries, however, the ability to quantify the accuracy of species identification with DNA barcoding is confounded. Correct identification is relative to some “gold standard”, itself subject to possible errors, so the standards themselves often need to be subject to scrutiny. Many papers on barcoding methodology choose to focus on issues other than the species concept, e.g., on the quality of primer annealing to DNA, the different strategies for matching an unknown query sequence with sequences in a reference database, the use of BLAST scores, etc. [4], but the species definition lies at the heart of DNA barcoding. For general reference sets of sequence data, however, investigators often turn to GenBank [9], where non-experts may have submitted the species annotations (e.g., [10]). In many cases, therefore, the estimated accuracy of a species identification tool based on DNA barcoding contains unquantified uncertainties about taxonomic identifications in GenBank or other DNA databases [11], and possibly even methodological errors [12]. Our previous study within the fungal family Xylariaceae [13] mentioned some of these issues, e.g., while discussing the advantages and limitations of sequence-based fungal identification [14]. Statistical criteria for flagging questionable species identifications could help clean taxonomic datasets and could even help taxonomists to delineate species boundaries. In addition, in difficult cases statistical tests provide objective evidence in support of subjective taxonomic judgments about species boundaries.

As a case study, we considered the fungal genus *Ramaria* (Gomphales; Basidiomycota, described in SI Appendix Text 1), which contains many species with poorly defined boundaries when based on morphological features alone. Accordingly, we collected a sequence dataset of the formal fungal barcode, ITS [15], including both GenBank and a separate set of specimens with expert taxonomic identification. Our dataset also included a few ITS sequences from genera close to *Ramaria*.

With the dataset in hand, while exploring taxonomy specific to *Ramaria*, we pursued the following general aims in DNA barcoding. First, bioinformatics pipelines in barcoding are often subject to “tweaking”, i.e., the pipelines undergo subtle variations intended to improve barcoding analyses. The probability of correct identification (PCI) is a figure of merit for comparing variant pipelines for taxonomic identification [15–17]. Several types of PCI appear in the barcoding literature, without specific names for the different types (e.g., [18]). The present paper therefore initiates a methodical study of PCI properties by bestowing names on four of them.

To avoid confusion, the reader should note at the outset that the four PCIs are not algorithms for assigning species, such as a k-nearest-neighbor match to a sequence database, or a Bayesian probability that a sample belongs to a given species, etc. A PCI provides a method for evaluating how well a species-identification algorithm agrees with a “gold standard” taxonomy. In fact, the *Ramaria* genus is narrow enough taxonomically that most computer algorithms for species identification by DNA sequence should usually agree. For simplicity, therefore, this article takes nearest-neighbor matching as its species-identification algorithm. This article then examines in different scientific contexts, which of the four variant PCIs is the best figure of merit for evaluating the algorithm. The choice of the figure of merit is important, because it will evaluate progress as scientists develop algorithms for taxonomic identification.

The Theory section considers identification at the species level, although its ideas apply to other taxonomic levels. It contains formal mathematical definitions for the four PCIs, but the following set-up defines them informally for non-mathematical readers.

To begin, consider a species identification pipeline consisting of: a reference database of taxonomic sequences, a set of query sequences derived from taxonomic samples, and an algorithm for identifying a sample’s species. Several figures of merit (e.g., PCI, or perhaps the fraction of query sequences correctly identified, etc.) can quantify the accuracy of species identification. This article proposes several desirable properties that an appropriate figure of merit should possess.

As a simplifying approximation, our analysis assumes that for each query, the algorithm returns a list of equally likely species. The species list may contain the same species several times, or it may contain one or zero species. If it contains zero species, query identification has failed. If the list contains only the correct species, we say identification is unanimous (i.e., unanimously correct).

Many practical algorithms (e.g., species identification by nearest database neighbor) satisfy the assumption. In practice, even if an identification algorithm returns a probability or score with each species identification for a query (e.g., [19]), it might still accord with the assumption, if it were followed by a post-processing step that decided for each species identification, “yes or no?”

The following defines informally the four PCIs for a given species. The Theory section shows that they correspond to progressively more optimistic definitions of correct identification within a species. To begin, for each species under scrutiny, determine whether it has a barcode gap, i.e., whether its maximum intraspecies distance is strictly less than its minimum interspecies distance. The Barcode Gap species PCI is then either 1 or 0, depending on whether a barcode gap exists. Second, determine whether every query from a species under scrutiny produces an unanimously correct list of species. Accordingly, the Unanimous species PCI is then either 1 or 0. Third, determine the fraction of queries from a species under scrutiny produces an unanimously correct list of species. The fraction lies between 1 and 0, inclusive, and it is Average Unanimous species PCI. Fourth and finally, for each query from a species under scrutiny, determine the fraction of species in the output list that are the query’s species. Average the fractions for each query in a species to derive the Fractional species PCI.

Given a species identification algorithm, each of the four PCI measures (Barcode Gap, Unanimous, Average Unanimous, and Fractional) is a potential measure of its taxonomic accuracy. This article examines the strengths and weaknesses of each measure.

After considering each species PCI, the Consortium for the Barcode of Life Plant Working Group used the Barcode Gap PCI to select rbcL and matK as botanical barcode markers [16]; its Fungal Working Group followed by using the Barcode Gap PCI to select ITS as a mycological barcode marker [15]. Each of the four PCIs may be appropriate to different purposes, however. We show, e.g., that despite its use in selecting the formal barcode markers, the Barcode Gap PCI has some undesirable properties for detecting subtle improvements in barcoding pipelines for species identification.

Second, an investigator may wish to know when problematic taxonomy (e.g., non-expert species identification) or technical difficulties (e.g., truncated sequences) have intervened to lower PCIs spuriously. Based on its study of the Average Unanimous PCI, this article derives an objective criterion, a p-value based on an accepted statistical test [20], to flag species identifications that are problematic relative to an accepted reference or to detect systematic technical difficulties.

Third, sequence databases with taxonomic annotations are growing at unprecedented rates. Ideally, an estimated PCI predicts future accuracies of species identification, and it fails as a predictor, if its value changes drastically as a database grows. By resampling our database to produce smaller databases with a variant of the bootstrap [21]), we simulate how the four different PCIs change as a taxonomic database grows, showing that some PCIs are better than others in predicting their future values as a database grows. The Discussion points out that all four PCIs have another desirable property, taxonomic normalization, which compensates for datasets with overrepresented species. Some other measures of the accuracy of species identification, e.g., fraction of correctly identified query sequences, lack taxonomic normalization.

The article has the usual Results, Discussion, and Materials and Methods sections. In addition, a Theory section precedes our Material and Methods. The Theory section is not necessary to the detailed understanding of the rest of the article, but its mathematical results are universally applicable across taxonomy. It also describes a statistical test, a loose taxonomic analog of the log-rank test in the analysis of survivorship in clinical trials. The statistical test has many possible uses in taxonomy, e.g., it can decide whether species identification by taxonomic experts is significantly more accurate than inexpert identification.

To delineate the taxonomic species boundaries in *Ramaria*, we collected ITS nrDNA for comparison with orthologous sequences from GenBank and UNITE databases. While contrasting the utility of each PCI variant in different contexts, we evaluated the putative taxonomy of samples using formal statistics to augment DNA barcoding with parsimony and Bayesian phylogenetic analyses. The indistinct species boundaries make the dataset a good test case for quantifying the possible influence of uncertain taxonomy, incomplete species boundaries, and other factors (e.g., DNA sequence length and the presence of ambiguous nucleotides) on the apparent accuracy of DNA barcoding, as measured by different PCIs.

## Results

### Morphological analyses

**Fig 1:**
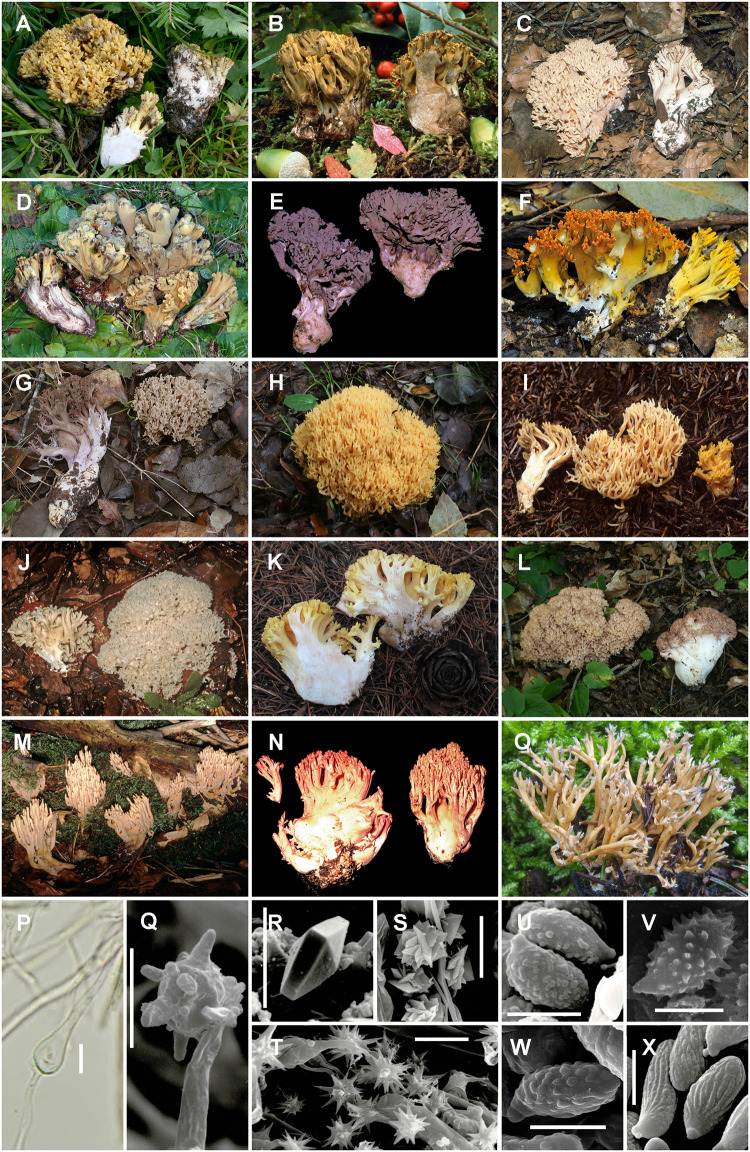
Morphological diversity of *Ramaria* species: (A) *R*. *abetonensis* AH 48006; (B) *R*. *arcosuensis* MA-Fungi 48049; (C) *R*. *bataillei* MA-Fungi 49453; (D) *R*. *broomei* MA-Fungi 79903; (E) *R*. *cedretorum* MA-Fungi 48074; (F) *R*. *cokeri* MA-Fungi 79893; (G) *R*. *fennica* var. *fumigata* AH 47767; (H) *R*. *flavescens* AH 48029; (I) *R*. *ignicolor* MA-Fungi 47978; (J) *R*. *mediterranea* MA-Fungi 39877; (K) *R*. *praecox* AH 47804; (L) *R*. *rubrievanescens* AH 47481; (M) *R*. *stricta* MA-Fungi 48068; (N) *R*. *subbotrytis* MA-Fungi 48088; (O) *R*. *subdecurrens* AH 48370; (P) Ampulliform septum of *R*. aff. *capucina* AH 48381; (Q) Cystidium on rhizomorphs from *R*. *quercus-ilicis* MA-Fungi 47984; (R) Bipyramidal crystal on tomentum from *R*. *mediterranea* MA-Fungi 39877; (S) Rosette crystals on rhizomorph hyphae from *R*. *comitis* MA-Fungi 47970; (T) Star-shaped crystals on rhizomorph hyphae from *R*. *flaccida* MA-Fungi 48020; (U) Spores of *R*. *comitis* MA-Fungi 47970; (V) Spores of *R*. *cokeri* MA-Fungi 79893; (W) Spores of *R*. *praecox* AH 47732; (X) Spores of *R*. *botrytis* MA-Fungi 48056. Scale bar = 5 μm.

Fig 1 illustrates the morphological diversity of *Ramaria* species. S1 Text describes the morphometric analyses.

### Sequence analyses

Our Total dataset contained 647 ITS sequences, with 231 sequences as “*Ramaria* sp.” S1 Table lists the PCI dataset used in the barcode analysis, which contained the remaining 416 sequences, with 382 unique sequences and 416–382 = 34 replicates. Our Long dataset was the subset of the PCI dataset whose sequences had length at least 500 bp. It contained 381 sequences, with 347 unique sequences and 34 replicates. Our Short dataset was the complement of the Long dataset within the PCI dataset. Our Taxonomic dataset was the subset of the PCI dataset whose sequences had expert taxonomic species identification. It consisted of 153 samples (107 samples that we identified, plus 46 GenBank sequences annotated as species types), with 151 unique sequences and 2 replicates. S2 Text contains “Sequence analyses” with summary statistics of the sequence length and nucleotide compositions of subsets of the PCI dataset. S1 Table lists the PCI dataset highlighting its Taxonomic and Short subsets

### Barcode analysis

Call a sample or species a “singleton”, if the relevant dataset contains only a single DNA sequence from the species. The PCI dataset retained singletons, because they provide decoys that complicate the species identification of other samples, mimicking practical difficulties in barcoding [16]. To quantify barcoding accuracy, the present article defines four variants of the probability of correct identification (PCI), all of which are averages of species PCIs over non-singleton species. The Methods section describes the four PCIs, and the Theory section defines them mathematically.

The PCI dataset had 416 samples, distributed among 75 singletons and 71 non-singleton species. Of these 75 singletons, two were from the genus *Gloeocantharellus*; one, from the genus *Gomphus*; and one, from the genus *Schildia*. None of these singletons shared its sequence with another species. The PCI dataset also contained four samples from *Gomphus clavatus*; two, from *Gomphus ludovicianus*; and three, from *Turbinellus floccosus*, so these non-singleton species contributed to PCIs. *Ramaria* samples contributed all other Species PCIs for the PCI dataset.

Presented as mean ± one standard error mean, the PCIs averaged over the *n* = 71 non-singleton species in the PCI dataset were: the Barcode Gap PCI *p*_*B*_ = 0.324±0.056; the Unanimous PCI *p*_*U*_ = 0.451±0.059; the Average Unanimous PCI *p*_*A*_ = 0.691±0.040; and the Fractional PCI *p*_*F*_ = 0.731±0.038. On any fixed dataset like the PCI dataset, the PCI inequality 0≤*p*_*B*_≤*p*_*U*_≤*p*_*A*_≤*p*_*F*_≤1 always holds (as demonstrated by the mathematical “Proof of the PCI Inequality” in the Theory section). As the PCIs increase, they convey increasingly optimistic assessments of the same set of species identifications. Thus, the PCI inequality shows that the PCIs reflect progressively weaker criteria for correct species identification.

To assess annotations misidentifying species within the PCI dataset, we partitioned the PCI dataset into two non-overlapping sets: a Taxonomic dataset (153 samples, distributed among 74 singletons and 26 non-singleton species) and a GenBank dataset (263 samples). The GenBank dataset consisted of every GenBank sequence not annotated as having derived from a species typus. We then modified the log-rank test in survival analysis for taxonomic purposes (see details under “A Taxonomic Unanimity Test” in S2 Text). The data for the unanimity test are a series of 2x2 tables. In the context of survival analysis, each table represents a different time of observation, and the table for each time counts the patients in each of the control and treatment groups that survived or died since the previous observation. In contrast, the taxonomic unanimity test does not impose any temporal relationships among the 2x2 tables of the log-rank test. It retains, however, the formal mathematical manipulations of the log-rank test to produce a z-score with an approximately Gaussian distribution. In our taxonomic application, each table represents a different species, and it counts the samples in each of the Taxonomic and GenBank datasets that were correctly or incorrectly identified (i.e., the identifications agreed or disagreed with annotation). Applied to the 71 non-singleton species, the taxonomic unanimity test gave a continuity-corrected z-score *Z* = 1.055–0.260 = 0.795 (written as uncorrected z-score minus its continuity correction) with a one-sided p-value *p* = 0.213. If the p-value fell below a significance threshold, we could declare at that threshold that the Taxonomic dataset contained significantly better species identifications than the GenBank dataset. Within our PCI dataset, therefore, GenBank identifications were not significantly worse than expert taxonomy. The implicit species structure of the statistic suggests the explicit construction of S3 and S4 Tables in S2 Text, where barcoding results highlight possibly misannotated samples for further taxonomic scrutiny.

In comparing barcoding pipelines for species identification, the taxonomic unanimity test can also indicate when a pipeline significantly improves species identification. Short ITS sequences might have degraded species identification, so we considered removing them from our analysis pipeline. To assess the degradation, we partitioned the PCI dataset into a Long dataset (381 samples, distributed among 73 singletons and 67 non-singleton species) and a Short dataset (35 samples). The Short dataset contained every non-singleton sample sequence with length less than 500 bp. The resulting continuity-corrected z-score *Z* = 0.509–0.301 = 0.208 (again, written as uncorrected z-score minus its continuity correction) from the taxonomic unanimity test yielded a one-sided p-value *p* = 0.418. If the p-value fell below a significance threshold, we could declare at that threshold that the Long dataset contained significantly better species identifications than the Short dataset. The actual p-value shows that the estimated barcoding accuracy of species identification (using the fixed sampling density of the PCI dataset) did not differ significantly between the Short and Long datasets. Like S3 Table in S2 Text for the GenBank dataset, S4 Table in S2 Text lists the samples in the Short dataset, where barcoding results single out possibly misannotated samples for further taxonomic scrutiny.

Implicitly, the taxonomic unanimity test on the Short dataset above used the following bioinformatics pipeline *A&E* (“align and extract”): (1) perform a multiple sequence alignment of the PCI dataset; and then (2) extract the Long dataset while maintaining the original multiple sequence alignment. We had concerns, however, that pipeline *A&E* might be inferior and degrade species identification relative to another pipeline, *E&A*, which: (1) extracts the Long dataset first; and only then (2) aligns the extracted sequences. Accordingly, we performed a Wilcoxon matched-pair signed-rank test [22, p. 75–83] on the pairs of Species PCIs for each species from the two pipelines, *A&E* and *E&A*.

Table 1 shows the (Overall) PCIs under the headings Pipeline *A&E* and for Pipeline *E&A* (an Overall PCI is the average of Species PCIs over all species). The column headed by *n* counts the number of species where each pipeline produced a different Species PCI from the other (e.g., for the Fractional PCI *p*_*F*_, the pair of Species PCIs *p*_*F*,*s*_ was different for 18 species). None of the PCIs showed any statistical significance, so reusing sequence alignments from the PCI dataset did not significantly reduce the accuracy of species identification within the Long dataset. In fact, PCIs decrease from pipeline *A&E* to pipeline *E&A*, suggesting that reusing sequence alignments from the PCI dataset may be a better bioinformatics strategy than realigning subsets.

**Table 1:** Comparison of different pipelines on the LONG dataset.

PCI	Align & Extract Pipeline *A&E*	Extract & Align Pipeline *E&A*	Signed-rank Test	
			*n*	p-value
Fractional (*p*_*F*_)	0.769±0.036	0.768±0.037	18	0.915
Average Unanimous (*p*_*A*_)	0.725±0.037	0.722±0.038	16	0.450
Unanimous (*p*_*U*_)	0.522±0.061	0.493±0.061	6	1.000
Barcode Gap (*p*_*B*_)	0.418±0.060	0.403±0.060	3	1.000

Just as with the Long dataset, pipelines *A&E* and *E&A* may both be applied to the Taxonomic dataset. S5 Table in S2 Text gives results for the Taxonomic dataset in the same format as Table 1. In fact, the PCIs there increase from pipeline *A&E* to pipeline *E&A*, so the reuse of sequence alignments from the PCI dataset is not a consistently superior strategy.

**Fig 2:**
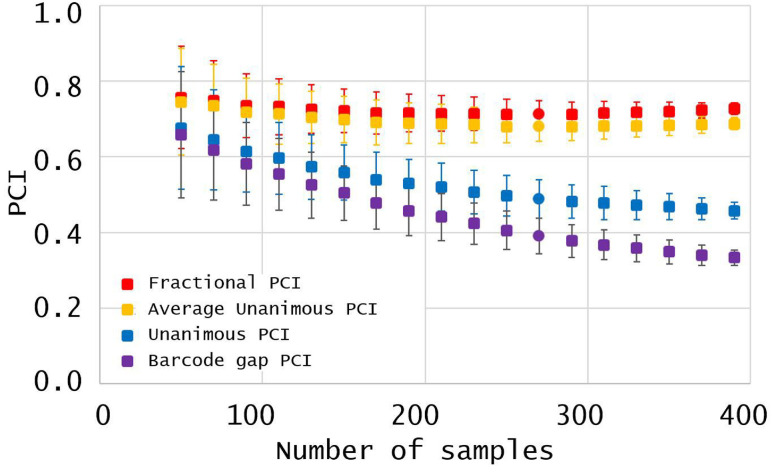
Resampling PCIs from the PCI dataset. The X-axis shows the number of samples in the dataset after resampling; the Y-axis, the probability of correct identification (PCI), with the points indicating mean and sample standard deviation of the resampled distribution. The PCIs are the Barcode Gap PCI *p*_*B*_ (bottom, purple), the Unanimous PCI *p*_*U*_ (second from bottom, blue), the Average Unanimous PCI *p*_*A*_ (second from top, orange), and the Fractional PCI *p*_*F*_ (top, red).

To quantify the effect of database size on our four PCIs, we resampled subsets of different sizes from the PCI dataset under uniform sampling without replacement (for details, see “Resampling the PCI dataset” in S2 Text). In Fig 2, the squares correspond to average PCIs in the resampled datasets of 50, 70, 90, …, 390 samples. Error bars display the sample standard error of each resampled PCI. They decrease to 0 as the resampled size approaches the size of the PCI dataset (416) because sampling without replacement becomes less random (e.g., at a size of 416, resampling without replacement simply selects the PCI dataset every time). Fig 2 complies with the PCI inequality 0≤*p*_*B*_≤*p*_*U*_≤*p*_*A*_≤*p*_*F*_≤1 and displays a partition of the PCIs into two groups: (1) the Fractional PCI *p*_*F*_ and the Average Unanimous PCI *p*_*A*_; and (2) the Unanimous PCI *p*_*U*_ and the Barcode Gap PCI *p*_*B*_. Compared to *p*_*U*_ and *p*_*B*_, *p*_*F*_ and *p*_*A*_ remain relatively constant as the resampled dataset increases in size; moreover, their error bars exhibit progressively less random variation.

During analysis, the PCI dataset underwent a small revision (with S1 and S2 Tables giving the final datasets). After completion of the barcode analysis (with results given verbatim above), e.g., the species *Ramaria rubribrunnescens* provided a concrete example of the effects of misannotation on barcode analysis. All its six samples in the PCI dataset were from GenBank. We discovered, however, that another GenBank sample (KY354750) was in fact from *Ramaria rubribrunnescens* and had been misannotated as *Ramaria rubribru**n**escens*. Under barcode computations, therefore, it was a singleton sample from a non-existent species. KY354750 shared its sequence with JX310406 (also *Ramaria rubribrunnescens*), and together, their identical sequences provided the two nearest neighbors of EU652352 (also *Ramaria rubribrunnescens*). In fact, barcode species identification within *Ramaria rubribrunnescens* was perfect, i.e., the species PCIs for *s* = *Ramaria rubribrunnescens* were *p*_*B*,*s*_ = *p*_*U*,*s*_ = *p*_*A*,*s*_ = *p*_*F*,*s*_ = 1 (see “Mathematical Definitions of the Four PCIs” in the Theory section). The misannotated sample, however, had spuriously reduced the species PCIs to *p*_*B*,*s*_ = *p*_*U*,*s*_ = 0, *p*_*A*,*s*_ = 4/6≈0.667, and *p*_*F*,*s*_ = 4.5/6 = 0.750, with the species PCIs having the most stringent criteria for correct species identification, the Barcode PCI *p*_*B*,*s*_ and the Unanimous PCI *p*_*U*,*s*_, suffering the greatest distortion. The PCI dataset had 71 non-singleton species, however so the misannotation perturbed overall PCIs by at most (1–0)/71≈0.014, well within statistical errors (see the PCIs at the start of “Barcode analysis” in the Results).

### Supplementary parsimony and Bayesian analyses

**Fig 3:**
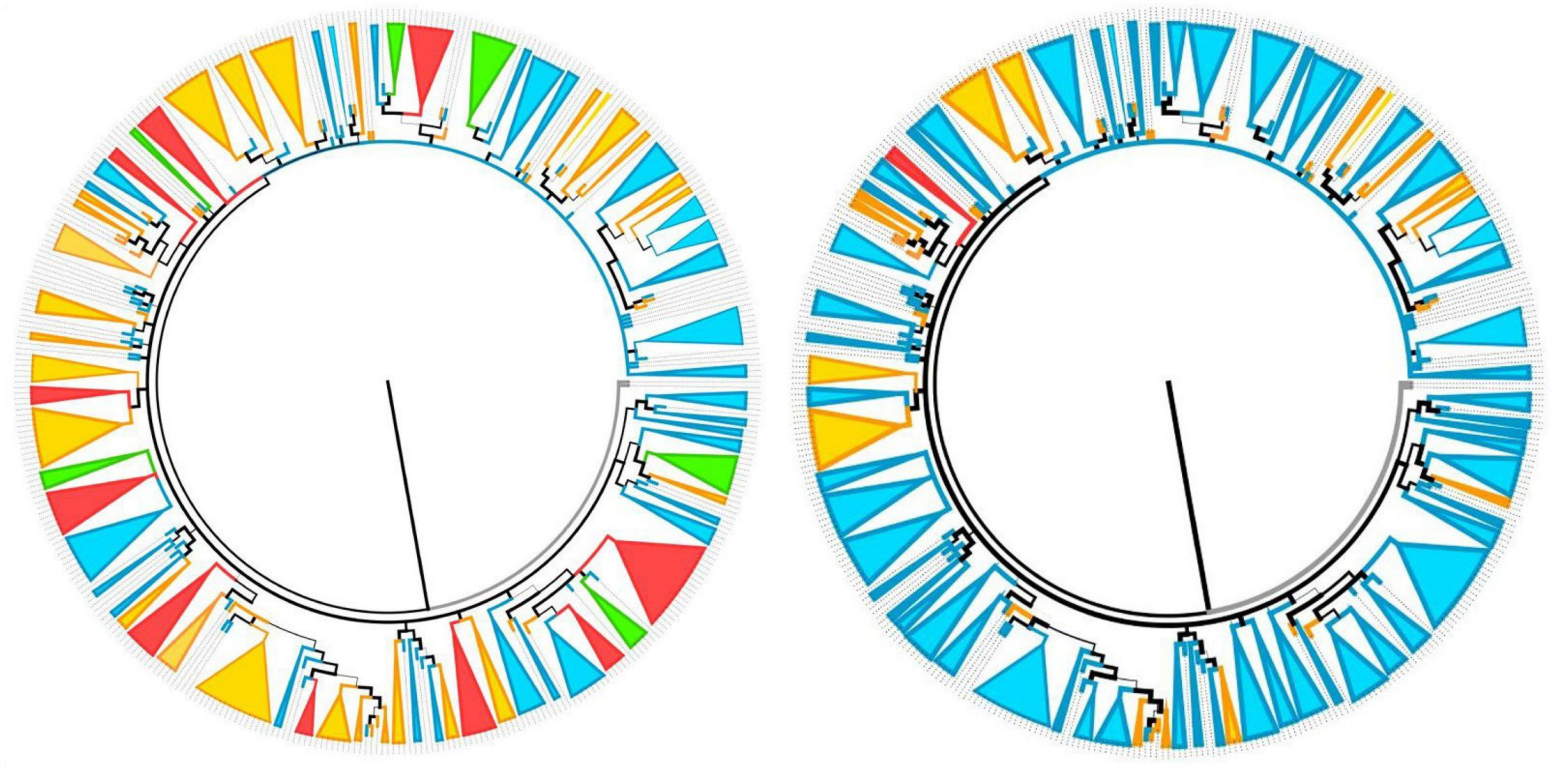
The 50% majority rule Bayesian tree inferred from ITS nrDNA assuming HSK+ I + G model of Gomphales included in the PCI analyses. Colors: Blue, unique species; Green, one species and related infraspecific taxa; Red, clades including two or more species; Orange: species that occur in two or more clades. See S1 Table and S1 Fig for clades and singleton names. In Fig 3, the left circle corresponds to clade identifications before DNA barcoding; the right, after DNA barcoding. Essentially, therefore, the left circle displays morphological species; the right circle displays the clades after DNA barcoding has increased the apparent number of monophyletic species.

S1 and S2 Tables list the datasets contributing to the phylogenetic analysis (with the correct species annotation for KY354750). In our analyses (Fig 3 and S1 Fig), including two *Gloeocantharellus* as outgroup, 73 clades and 74 singletons were obtained, of which 97 samples could be assigned to known taxa (3 *Gomphus*, 92 *Ramaria*, 1 *Turbinellus*, and 1 *Schildia*), and 50 might be undescribed species, but also possibly known species but misidentified, or related to known taxa (e.g. *R*. *conjunctipes*-clade 2, *R*. *gracilis* 1, *R*. *thiersii* 1, *R*. *neoformosa* 1).

As shown in Fig 3 and S1 Fig (the same tree with sample identifiers at its leaves), 31 clades and 50 singletons (in blue) represent single species; 5 clades (in green) included related infraspecific taxa; 10 clades (in red) included sequences with different species; and 27 clades and 24 singletons (in orange) belong to 23 taxa that occur in two or more clades.

Some examples of clades with only one species (S1 Fig, in blue) are *Ramaria suecica*, *R*. *stuntzii*, *R*. *maculatipes* or *R*. *amyloidea*; and the singletons R. *cyanocephala*, *R*. *boreimaxima* or *R*. *quercus-ilicis*. In the case of green clades, such as *R*. *flavescens*, the sequence obtained from the type of *R*. *flavescens* var. *suaveolens* is included; the other four green clades also include a species and its varieties. The most significant example of red clades is in the top of S1 Fig, and involve seven names (*Ramaria abietina*, *R*. *abietina 1*, *R*. *apiculata*, *R*. *apiculata 1*, *R*. *apiculata* var. *brunnea*, *R*. *rubella*, and *R*. *tsugina*). In the case of orange clades, a well-known species in temperate regions, such as *R*. *abietina*, appears not only in the red clades already mentioned, but also in the red clades named by us as *R*. *flaccida* and two orange clades, one of them the *R*. *abietina* sensu stricto. The right of Fig 3 displays how information from DNA barcoding improved tentative initial morphological identifications shown on the left of Fig 3 to increase clade consistency, particularly in the final clades belonging to a single species (in blue).

Of the 35 short sequences included in the analysis, 24 matched with expected clades (singletons excluded); and of 12 with ambiguous nucleotides, only one matched with expected clades (*R*. *argentea* JQ408231 from GenBank).

## Discussion

### Barcode analysis

Typically in DNA barcoding, probabilities of correct identification (PCIs) provide the figure of merit for comparing variant bioinformatics pipelines [17]. The barcoding literature contains several types of PCI (e.g., [18]), and unjustifiably, some studies have even compared different types of PCIs directly, as though their status as probabilities permitted direct comparison. The PCI Inequality 0≤*p*_*B*_≤*p*_*U*_≤*p*_*A*_≤*p*_*F*_≤1, proved mathematically shows that for a fixed dataset, some types of PCIs are always larger than others. The type of PCI used is therefore critical in evaluating the results of a study.

PCIs similar to species PCIs can be calculated at other taxonomic levels like genus, family, etc. As figures of merit for taxonomic identification, the corresponding overall PCIs have the property of “taxonomic normalization”, described here only for the species level. In contrast, several studies [19, 23–25] used a measure related to PCI, namely, the fraction of sequences with correctly identified species. To demonstrate by exaggeration the defects of the measure, if 75% of samples come from a single species, the figure is thoroughly skewed by the dominant species. On the other hand, an overall PCI that averages species PCIs automatically normalizes datasets against overrepresented species [26]. Sometimes, normalization may be undesirable, e.g., if species have differing importance to the purpose at hand, an explicitly weighted average of species PCIs may be pertinent. Regardless, the ability of the overall PCI to normalize datasets taxonomically and reduce skewing by over-represented species led to its broad acceptance among botanists [16] and mycologists [15].

Taxonomic normalization has other desirable consequences. Measures without it require detailed ancillary data to support their relevance, e.g., rather than just a species count or enumeration, those measures require a separate demonstration that the sequences are about evenly distributed across the relevant species. When comparing different datasets, a figure of merit requiring detailed ancillary data can seriously impede human evaluation (e.g., Section 3.2 in [27] criticizes the ROC_*n*_ in sequence retrieval because the ROC_*n*_ requires ancillary information to verify its validity). To avoid distracting humans with detailed ancillary data, the figure of merit then requires a rigid dependence on benchmarking with standard datasets to ensure scientific progress.

As indicated in the Introduction and Fig 2, a figure of merit should remain stable as a database grows. If adventitious database features like species overrepresentation can profoundly alter a figure of merit, the figure is not fulfilling its purpose.

For any specific purpose, the four types of PCI may have widely differing utilities. The Barcode Gap PCI (*p*_*B*_) reflects the most stringent criterion for correct species identification, namely, the presence or absence of a barcode gap for a species. Because of its stringency, and because DNA barcodes might be used to construct phylogenetic trees as well as to identify species, botanists [16] and mycologists [15] used the Barcode Gap PCI to select their barcode markers. Table 1 indicates that compared to other types of PCIs, the Barcode Gap PCI is less broadly informative of species behavior in a bioinformatics pipeline, however. The column labeled *n* in Table 1 shows that only three species had different Species Barcode Gap PCIs for pipelines *A&E* and *E&A*. As criteria for correct species identification became less stringent (i.e., as PCIs became larger), *n* also increases, reaching a maximum value *n* = 18 for the Fractional PCI. S5 Table in S2 Text of the SI displays a similar trend.

The Barcode Gap PCI displays impressive improvements in Table 1 and S5 Table in S2 Text of the SI, but a single misannotated sample can spuriously destroy the barcode gap in a species *s*. The Results for *Ramaria rubribrunnescens* show that a single sample can change the PCI for species *s* violently, from *p*_*B*,*s*_ = 1 to *p*_*B*,*s*_ = 0, so the Barcode Gap PCI displays an undesirable sensitivity to errors in the data. The Unanimous PCI (*p*_*U*_) suffers a similar defect, because all samples in a species *s* are either correctly identified or not (i.e., either *p*_*U*,*s*_ = 1 or *p*_*U*,*s*_ = 0).

Insensitivity to subtle changes across several species and vulnerability to misannotation or otherwise anomalous taxonomy probably make the Barcode Gap and Unanimous PCIs among the worst PCIs for assessing gradual improvements in a barcoding pipeline. After the proof of the PCI inequality, the Theory section contains a second mathematical proof: if the Unanimous PCI *p*_*U*,*s*_ can detect that procedural pipeline has improved identification within a species *s*, the Average Unanimous PCI *p*_*A*,*s*_ can also. The statement virtually disqualifies the Unanimous PCI as a desirable figure of merit when improving barcoding pipelines.

A violent discontinuity in a PCI has other undesirable consequences. Fig 2 shows results from resampling the PCI dataset to produce smaller datasets. Resampling (e.g., the bootstrap [28]) is a popular method to make inferences about complex situations, although its inferences about maxima and minima carry some caveats (see particularly, counter-example 2 in [29]). When querying a barcode database with a new sample, many practical computer algorithms for species identification depend on the new sample’s nearest neighbors, database sequences that minimize some distance to the sample sequence. This article measured genetic variation between two aligned DNA sequences with p-distance [30], the fraction of unambiguous nucleotide pairs that were mismatches. Caveats notwithstanding, Fig 2 indicates that the present values of *p*_*B*_ and *p*_*U*_ are less predictive than *p*_*A*_ and *p*_*F*_ of their future values as a barcoding database grows. As new samples enter a barcoding database, a single sample in a species *s* can reduce the Barcode Gap or Unanimous PCI for species *s* (*p*_*B*,*s*_ or *p*_*U*,*s*_) violently, from 1 to 0, whereas its effects on the other PCIs are more gradual (e.g., see again the Results for *Ramaria rubribrunnescens*).

When evaluating a pipeline for taxonomic identification, a singleton species may cause difficulties if only one sequence dataset must serve both as a reference database and a source of queries. Following the pattern of previous studies [15, 16], our PCIs considered only non-singleton species and omitted each query in turn from the reference database. Each query then had a species representative in the reference database available as a possible nearest neighbor. Consequently, the evaluation of the pipeline conferred a special status on singletons, not our identification algorithm. At first, the distinction between evaluating a pipeline and the pipeline itself can be subtle, and the reader should reread this paragraph if its point is unclear. The early paragraph in the Introduction, starting “To avoid confusion…” is also relevant.

When the evaluation of a pipeline uses a single dataset as a reference database and a source of queries, PCIs should be accompanied by a separate count of species singletons having unique sequences. There is nothing objectionable in counting the singletons separately, but calculating a single combined PCI from non-singleton species and the singletons [25] has the undesirable consequences mentioned during the discussion of resampling: violent change and lack of predictability as a barcoding database grows. In particular, a single PCI combined with singletons typically views a singleton as correctly identified if its sequence is unique within the database. Another sample from the same species entering the reference database then lowers the species PCI from 1 to 0, and no third sample from its species modulates the change.

On one hand, although we examined the genus *Ramaria* as a case study, we expect our observations to be most pertinent to other taxa with relatively low PCIs. On the other hand, if taxonomic identification is straightforward, then methodology becomes less important.

The present article also introduced a taxonomic unanimity test. The tests and tables of samples derived from the test (e.g., S3 and S4 Tables in S2 Text of the SI) may help when cleaning taxonomy from and reporting errors to a database like GenBank or allaying fears about using sequences with ambiguous nucleotides. Similarly, the signed-rank test applied species by species showed that expedients like reusing sequence alignments did not significantly worsen results. With other datasets, it might have shown that improvements to a barcoding pipeline are statistically significant. We chose to use it, because it has greater statistical power than its competitor, the sign test, making it an orthodox choice. In a taxonomic context, however, the signed-rank test gains its power by using ranks to break the symmetry of the species under scrutiny. Studies rarely sample species evenly, however, so sampling biases can subtly and systematically influence the ranking of species, and therefore the ranked-sign test. Sampling biases do not affect the sign test, which maintains symmetry between species throughout the analysis. In the taxonomic context, therefore, the sign test has some features to recommend it, but its use would not have changed the scientific conclusions in this article.

In addition, although not performed here because of insufficiently deep sampling of two closely related species, a Fisher exact test for individual 2x2 tables (two species, two experts) could flag statistically significant expert disagreement on species boundaries, prompting closer examination of systematic expert disagreement on characters defining the species. The basic motivation for all statistical tests suggested here is that non-parametric tests [31] are readily applied to taxonomic groups of equal weight (here, each species PCI contributes equally to the p-value).

Statistical findings were uniformly negative within *Ramaria*. Within the PCI dataset, barcoding species identification was not significantly worsened by: (1) non-expert annotations in GenBank; or (2) short sequences (less than 500 bp). For both the Taxonomic and Long datasets, the pipeline reusing the sequence alignment from the PCI dataset did not have significantly worse species identification than the pipeline that extracted the datasets and then aligned them. The negative statistical findings therefore allayed our initial concerns that the PCI dataset required further filtering before barcoding analysis. In addition, they confirmed that the difficulties that *Ramaria* present to taxonomists extend to the nucleotide realm.

### Parsimony and Bayesian analyses

About a third part of the accepted *Ramaria* species (96/300) were included in our analyses, and 50 clades could be related to cryptic, misidentified or unknown species. As in other groups, some of them are known species, but they do not appear with the correct name in GenBank: in 2003, about 20% of the named sequences were misidentified [7, 32, 33]; more recently, nearly 30% [14]. In the opposite case, some sequences could appear misidentified, because they belong to different ITS copies of the same species; this was one the criticisms of using ITS as a universal barcode for fungi [34]. If species annotations are correct, ITS sequences from *Ramaria conjunctipes*, *R*. *botrytis*, *R*. *formosa* or *R*. *stricta* display a large molecular variation; and their clades could be species complexes or contain cryptic species.

*Ramaria* and related genera exemplify the importance of sound taxonomic knowledge before starting molecular analyses in a group. As a case study, they also show how molecular analysis helps taxonomists to improve the identification of doubtful samples, and how “the taxonomic feedback loop” links both disciplines synergistically [35]. During the last years, phylogenetic analyses using ITS sequences have helped us to elucidate many groups of fungi (e.g. gasteroid and corticioid) and to describe new species (e.g. [36–38]). Unfortunately, however, even if molecular analysis detects a new species, some authors do not follow through by describing and naming it. Moreover, they also avoid the responsibility of curating their sequences in DNA repositories as well as in publications. These repositories contain a many unidentified sequences, even though many of them are connected to vouchers and are not environmental samples. In our study, 647 sequences from Gomphales, mainly *Ramaria*, were downloaded, but 231 had a non-specific epithet (e.g., “sp.”), and our PCI analysis had to exclude them. Although not shown in this paper, many of these *Ramaria* sp. could be assigned to a known species, however. On one hand, GenBank places sample KT824242 under *Ramaria* sp. (S2 Table), e.g., but its specimen voucher (KD-14-006) is the holotype of *R*. *subalpina* [39]. On the other hand, sample MH216040 (S2 Table, holotype) was under *Ramaria* sp. PA-2018 when we did the analyses, but was later renamed as *R*. *parabotrytis* [40].

**Fig 4:**
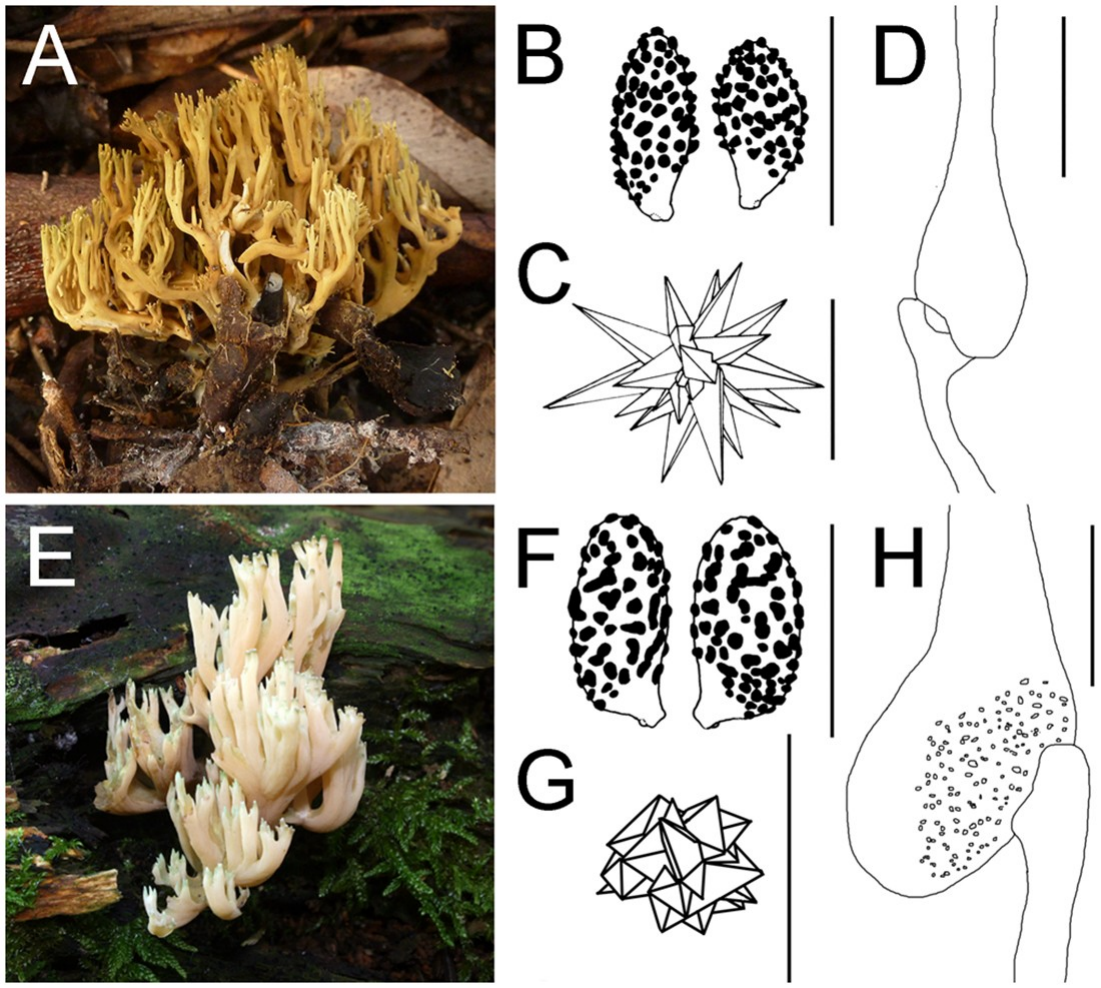
Comparative of features of *Ramaria abietina* and *R*. *apiculata*. *Ramaria abietina*: (A) Basidioma (AH 48373); (B) Spores (MA-Fungi 49419); (C) Crystals from mycelium (MA-Fungi 49419); (D) Ampulliform septum (MA-Fungi 49419). *Ramaria apiculata*: (E) Basidioma (AH 47751); (F) Spores (MA-Fungi 48462); (G) Crystal from mycelium (MA-Fungi 48485); (H) Ampulliform septum (MA-Fungi 47981). Scale bars = 10 μm.

Many species names appear to be added after a direct BLAST search, identified only by macromorphology, without microscopic examination, e.g., *R*. *apiculata* clade contains many sequences under *R*. *abietina*. Microscopical features differentiate the two species: *R*. *apiculata* has rosette oxalate crystals in the mycelium, ellipsoid and verrucose spores, and ornamented ampulliform septa, whereas *R*. *abietina* has stellate oxalate crystals in the mycelium, amygdaloid and nearly spinose spores, and smooth ampulliform septa (Fig 4). This is a good example showing that DNA repositories need to include high quality sequences from well identified specimens [41, 42].

**Fig 5:**
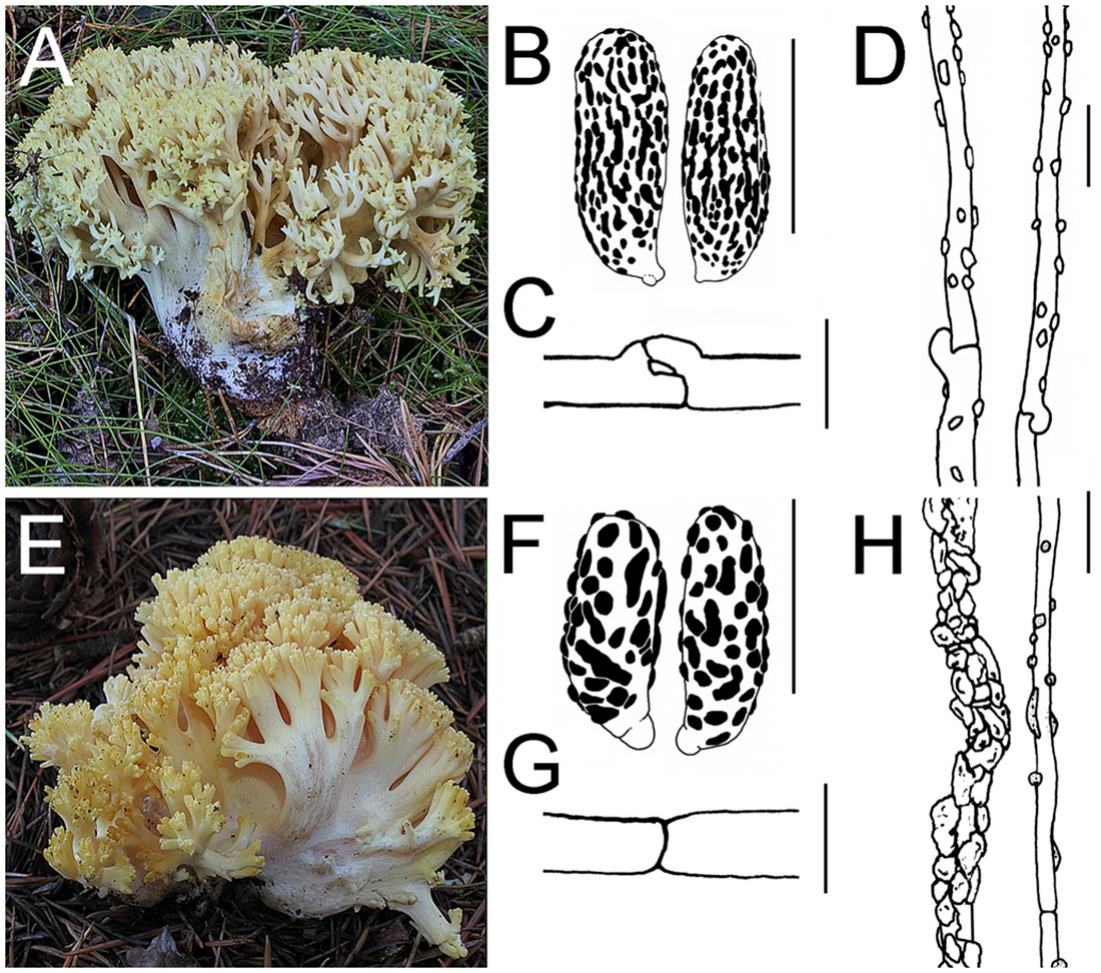
Comparative of features of *Ramaria flava* and *R*. *praecox*. *Ramaria flava*: (A) Basidioma (AH 48375); (B) Spores (MA-Fungi 48465); (C) Clamped hyphae from context (MA-Fungi 48484); (D) Hyphae from mycelium (MA-Fungi 48484). *Ramaria praecox*: (E) Basidioma (AH 47804); (F) Spores (AH 47732); (G) Unclamped hyphae from context (AH 47732); (H) Hyphae from mycelium with crystals (AH 47804) or mucilaginose drops (AH 47732). Scale bars = 10 μm.

In addition, many mistakes in the DNA repositories are related with contaminations or mixed-up samples. Among the sequences obtained for this study, after the phylogenetic analyses we detected a contamination; our sequence AF442098 identified as *R*. *praecox* grouped in the *R*. *flava* clades. However, *R*, *praecox* is very different to *R*. *flava*. In *R*. *praecox* spores show conspicuous ornamentation, hyphae without clamps, and vernal phenology, whereas in *R*. *flava*, spores are slightly ornamented, hyphae clamped, and mainly autumnal phenology (Fig 5). Just as in metagenomic sequencing [43], contamination can arise from previous amplifications. Here, although we also include negative controls to each set of amplifications as a routine, contamination occurred.

A preliminary analysis detected possible mix-ups in the *R*. aff. *spinulosa* sequence and one sequence of *R*. *botrytis*, so we amplified and sequenced them again. The sequences were then correct: AF442904 corresponded to *R*. aff. *spinulosa*; and AJ292294, to *R*. *botrytis*. Only taxonomic characters of the species can expose such errors, so the metadata in DNA repositories should include the voucher for sequences [44]. (*R*. *botrytis* has grooved spores from a fleshy and white basidioma with reddish apices whereas *R*. aff. *spinulosa* has verrucose spores from a delicate brownish basidioma with concolor apices).

Moreover, we must remember questions related to nomenclature, e.g., sequences in DNA repositories that belong to one species, but appearing under two or more different names. During our morphological study we have unified some of these names. Thus, for example, *R*. *luteovernalis* is synonymous with *R*. *praecox*; or *R*. *fennica* var. *violacea* synonymous with *R*. *cedretorum* (S1 Table). In other cases (S1 Fig and S1 Table), some nomenclature questions to be addressed after the phylogenetic analyses included in this study, e.g., *R*. *claviramulata* and *R*. *celerivirescens* published as new species in [45], where our analyses grouped them into the same clades. Exeter et al. [46] suggested that *R*. *celerivirescens* is the correct name to the species. We studied the unique collection from *R*. *claviramulata* (holotype), and the basidiomata show a morphology that could correspond with a phytoplasma infection, as we have shown in other *Ramaria* species [47].

## Theory

In the main article, formal mathematical definitions and proofs are restricted to this Theory section, which is not necessary to understand the rest of the article. The Theory section defines the four PCIs mathematically, proves the PCI inequality, and proves that Average Unanimous PCI *p*_*A*,*s*_ is more sensitive than the Unanimous PCI *p*_*U*,*s*_ for detecting improvements in a procedural pipeline. It also introduces a taxonomic unanimity test for detecting statistically significant improvements in barcode species assignment, e.g., to reflect expert species identification or pipeline improvements.

### Mathematical definitions of the four PCIs

Fix a species *s*, with *n* samples in *s*. For each sample *σ* = 1, 2,…,*n* from *s*, define the nearest neighbors of *σ* to have the minimum p-distance from *σ* (in the relevant dataset). Let the fraction of nearest neighbors of *σ* within *s* be *f*_*σ*_(0 ≤ *f*_*σ*_ ≤ 1). Define the unanimity function *U*(*f*_*σ*_) = 1 if *f*_*σ*_ = 1 (all nearest neighbors of *σ* are in *s*); and 0, otherwise. For species *s*, define the Species Fractional PCI *p*_*F*,*s*_ = (*f*_1_ + *f*_2_ +…+ *f*_*n*_)/*n*; the Species Average Unanimous PCI *p*_*A*,*s*_ = (*U*(*f*_1_)+*U*(*f*_2_)+…+*U*(*f*_*n*_))/*n*; and the Species Unanimous PCI *p*_*U*,*s*_ = min{*U*(*f*_1_), *U*(*f*_2_),…,*U*(*f*_*n*_)}. The Species Barcode Gap PCI *p*_*B*,*s*_ = 1 if the maximum intraspecies p-distance is strictly less than the minimum interspecies distance (i.e., species *s* has a barcode gap); and 0, otherwise. Note for any species *s*: (1) *p*_*U*,*s*_ and *p*_*B*,*s*_ only take the values 0 and 1; and (2) all four of the Species PCIs *p*_*s*_ ∈ (*p*_*F*,*s*_, *p*_*A*,*s*_, *p*_*U*,*s*_, *p*_*B*,*s*_) satisfy 0 ≤ *p*_*s*_ ≤ 1. For any of the four Species PCIs *p*_*s*_, define a corresponding (Overall) PCI *p* by averaging the Species PCI over all species. In a standard notation from physics, we denote the average over species with angle brackets, e.g., *p*_*B*_ = 〈*p*_*B*,*s*_〉_*s*_. S1 Table contains several examples of these calculations for samples in species in the PCI dataset.

### Proof of the PCI inequality

$$0\leq p_{B,s}\leq p_{U,s}\leq p_{A,s}\leq p_{F,s}\leq 1.$$ (1)

By definition, 0≤*p*_*B*,*s*_. To prove *p*_*B*,*s*_ ≤ *p*_*U*,*s*_, there are two cases: *p*_*B*,*s*_ = 0 or *p*_*B*,*s*_ = 1. If *p*_*B*,*s*_ = 0, then automatically *p*_*B*,*s*_ ≤ *p*_*U*,*s*_. Otherwise, *p*_*B*,*s*_ = 1, and the barcode gap for species *s* implies that for every sample *σ* in the species *s*, all nearest neighbors belong to *s*, i.e., *f*_*σ*_ = 1 and *U*(*f*_*σ*_) = 1. Thus, *p*_*U*,*s*_ = min{*U*(*f*_1_), *U*(*f*_2_), …,*U*(*f*_*n*_)} = 1, so again *p*_*B*,*s*_ ≤ *p*_*U*,*s*_. To prove *p*_*U*,*s*_ ≤ *p*_*A*,*s*_, the minimum *p*_*U*,*s*_ of the numbers *U*(*f*_*σ*_) can never exceed their average *p*_*A*,*s*_. To prove *p*_*A*,*s*_ ≤ *p*_*F*,*s*_, note that *U*(*f*_*σ*_) ≤ *f*_*σ*_ (on one hand, if *f*_*σ*_ = 1, then *U*(*f*_*σ*_) = *f*_*σ*_ = 1; on the other hand, if *f*_*σ*_ < 1, then 0 = *U*(*f*_*σ*_) ≤ *f*_*σ*_). Average over all samples in the species *s* to derive

$$p_{A,s}=(U(f_{1})+U(f_{2})+\ldots +U(f_{n}))/n\leq (f_{1}+f_{2}+\ldots +f_{n})/n=p_{F,s}.$$ (2)

Finally, *p*_*F*,*s*_ ≤ 1 to finish the proof of Eq (1). In Eq (1), average over all species *s* to derive

$$0\leq {\langle p_{B,s}\rangle }_{s}\leq {\langle p_{U,s}\rangle }_{s}\leq {\langle p_{A,s}\rangle }_{s}\leq {\langle p_{F,s}\rangle }_{s}\leq 1.$$ (3)

The definitions *p*_*B*_ = 〈*p*_*B*,*s*_〉_*s*_, etc., yield

$$0\leq p_{B}\leq p_{U}\leq p_{A}\leq p_{F}\leq 1,$$ (4)

finishing the proof.

### The Average Unanimous PCI *p*_*A*,*s*_ is more sensitive than the Unanimous PCI *p*_*U*,*s*_ for detecting improvements in a procedural pipeline

Consider a procedural pipeline (represented by unprimed symbols), later improved to another pipeline (represented by primed symbols). Then for any species *s*, if $p_{U,s}<{p^{\prime }}_{U,s}$, then $p_{A,s}<{p^{\prime }}_{A,s}=1$.

**Proof**: If $p_{U,s}<{p^{\prime }}_{U,s}$, then $0=p_{U,s}<{p^{\prime }}_{U,s}=1$ (because *p*_*U*,*s*_ is either 0 or 1). After improvement on one hand, ${p^{\prime }}_{U,s}=\text{min}\{U({f^{\prime }}_{1}),U({f^{\prime }}_{2}),\ldots ,U({f^{\prime }}_{n})\}=1$. Thus, $U({f^{\prime }}_{1})=U({f^{\prime }}_{2})=\ldots =U({f^{\prime }}_{n})=1$ and ${p^{\prime }}_{A,s}=(U({f^{\prime }}_{1})+U({f^{\prime }}_{2})+\ldots +U({f^{\prime }}_{n}))/n=1$. Originally on the other hand, *p*_*U*,*s*_ = min{*U*(*f*_1_), *U*(*f*_2_), …,*U*(*f*_*n*_)} = 0, so at least one *i* = 1, 2, …, *n* satisfied *U*(*f*_*i*_) = 0. Consequently, *p*_*A*,*s*_ = (*U*(*f*_1_) + *U*(*f*_2_)+…+*U*(*f*_*n*_))/*n*<1. Because ${p^{\prime }}_{A,s}=1$, we have proved $p_{A,s}<{p^{\prime }}_{A,s}=1$.

### A taxonomic unanimity test

A taxonomic unanimity test can detect improvements in barcode species assignment, as follows.

Consider a specific barcoding sequence dataset, in which every sequence bears a species identification from one of two sources. It might be useful to have objective evidence that one source (e.g., an expert taxonomist) is systematically more accurate than the other. In a related situation, one might be concerned that species identification is superior in one of two disjoint sets of samples, e.g., that sequences longer than 500 bp identify species better than sequences shorter than 501 bp. To be specific, consider the case of expert identification.

Consider a sequence dataset of interest and any species *s* with at least two samples in the dataset. The nearest neighbors of a sample from *s* are the sequences in the dataset, excluding the sample sequence itself, with the minimum p-distance to the sample sequence. We say that a sample from species *s* has unanimous neighbors if its nearest neighbors are all from *s*.

Let a dataset of *N* (more than 2) sequences be assembled from two sources, e.g., with and without expert species taxonomy. Construct a 2x2 table corresponding to the species *s* (see “A Taxonomic Unanimity Test” in S2 Text), as follows. Of the sequences with expert taxonomy, let *A* count the sequences with unanimous neighbors; *C*, the sequences without. Similarly, of the sequences without expert taxonomy, let *B* count the sequences with unanimous neighbors; *D*, the sequences without. Thus, of the *N* = *A*+*B*+*C*+*D* samples, *A*+*C* had expert species taxonomy, whereas *B*+*D* had not. Similarly, each sample within species *s* either had unanimous neighbors or had not, and of the *N* samples, *A*+*B* were unanimous, whereas *C*+*D* were not. One might hope that expert taxonomy displays itself by systematically increasing the number of unanimous neighbors.

If the unanimity of neighbors is probabilistically independent of the source of taxonomic identification, then in species *s*, of the *A*+*C* samples with expert identification, the expected count of samples with unanimous neighbors is *E*_*s*_ = (*A* + *C*)(*A* + *B*)/*N*, because (*A* + *B*)/*N* is fraction of samples with unanimous neighbors. The observed count of samples with expert taxonomy and unanimous neighbors is in fact *O*_*s*_ = *A*. Conditioned on the marginals *A*+*C*, *B*+*D*, *A*+*B*, and *C*+*D*, if the source of taxonomic identification is independent of the samples of species *s*, *A* follows a hypergeometric distribution, so its variance is

$$\sigma_{s}^{2}=\frac{\left(A+C)(B+D)(A+B)(C+D\right)}{N^{2}(N-1)}.$$ (5)

Sum the excess *O*_*s*_—*E*_*s*_ over all species *s* and denote the sum by *X*. Sum $\sigma_{s}^{2}$ similarly, and denote the result by *σ*^2^. Under the reasonable approximation that species are probabilistically independent, *X* has an approximate normal distribution with variance *σ*^2^. Thus, the upper tail of a standard normal cumulative distribution function beyond *Z* = *X*/*σ* gives a one-sided p-value for testing whether expert taxonomy is significantly more accurate than inexpert species identification.

## Materials and methods

S1 Text describes the morphological analyses, PCR, and sequencing of the fungal specimens studied in this paper.

### Sequence datasets

The Total dataset consisted of 647 sequences including those under *Ramaria* sp. in the GenBank. Moreover, *Gomphus*, *Schildia*, and *Turbinellus* sequences were included due to the relationship with *Ramaria* mentioned in the introduction. Two samples of *Gloeocantharellus* were chosen as outgroup to the posterior phylogenetic analyses. Before proceeding to the phylogenetic analyses, searches with BLAST+ 2.8.1 checked dubious sequences and genera misidentifications [48, 49]. The PCI dataset consisted of 416 sequences (specifically excluding *Ramaria* sp. in the Total dataset). S2 Text contains summary statistics describing the lengths and nucleotide compositions of the sequence data. To assess the effects of short ITS sequences on species identification, we subdivided the PCI dataset into two datasets: (1) the Long dataset, sequences exceeding 500 bp; and (2) the Short dataset containing the remaining sequences. To assess the effect of faulty taxonomy in GenBank sequences on species identification, we subdivided the PCI dataset into two datasets: (1) the Taxonomic dataset, our samples plus species type-derived sequences in GenBank; and (2) the GenBank dataset containing the remaining GenBank sequences.

### Multiple sequence alignment and p-distance

To prepare for multiple sequence alignment, we removed replicate sequences, so all aligned sequences were unique. Most multiple sequence alignment programs are progressive, so they add sequences sequentially and then align them. Progressive sequence alignment programs can therefore align replicate sequences differently, spuriously inferring a positive phylogenetic distance between identical sequences. In general, the removal of any replicate sequence before sequence alignment improves the stability of multiple sequence alignments [50], and surprisingly, the workflows in some multiple sequence alignment programs do not remove replicate sequences from their inputs before sequence alignment. Muscle 3.8.31 under its default parameters [51, 52] aligned our unique sequences in the Total, PCI, Long, and Taxonomic datasets.

The principle basic to DNA barcoding is that the genetic variation between species usually exceeds the variation within a species (e.g. [6, 53]). Many distances can measure genetic variation between sequences, but the choice of distance rarely influences species identifications (e.g., [13, 54–56]). For simplicity, the p-distance between two aligned DNA sequences [30], the fraction of unambiguous nucleotide pairs that were mismatches, measured genetic variation here.

### Four types of the probability of correct identification (PCI)

Many variants of PCI appear in the taxonomic literature (e.g., [18, 57]). Given a dataset, we calculated the following four types of Species PCIs for all non-singleton species *s*. (The Theory section contains a mathematically formal “Mathematical Definitions of the Four PCIs”).

The Species Barcode Gap PCI (*p*_*B*,*s*_) is 1 if species *s* has a barcode gap (i.e., its minimum interspecies p-distance exceeds its maximum intraspecies p-distance), and 0 otherwise.

Fix a dataset, and within species *s*, consider a sample’s nearest neighbors under p-distance. Call a sample unanimous (i.e., unanimously correctly identified) if its nearest neighbors under p-distance are all from species *s*. Each unanimous sample has a Sample Unanimous PCI of 1; all other samples have a Sample Unanimous PCI of 0. Define the Species Unanimous PCI (*p*_*U*,*s*_) as 1 if every sample in species *s* is unanimous, and 0 otherwise.

Averaging provides another procedure for turning Sample Unanimous PCIs into Species PCIs. Define the Species Average Unanimous PCI (*p*_*A*,*s*_) as the average of the Sample Unanimous PCIs over all samples in the species *s*.

Finally, let the Sample Fractional PCI to be the fraction of nearest neighbors belonging to species *s*. The Species Fractional PCI (*p*_*F*,*s*_) is the average of the Sample Fractional PCIs over all samples in species *s*.

Each of the four Species PCIs (*p*_*B*,*s*_, *p*_*U*,*s*_, *p*_*A*,*s*_, *p*_*F*,*s*_) may be averaged over all (non-singleton) species *s* to produce the corresponding (Overall) PCI (*p*_*B*_, *p*_*U*_, *p*_*A*_, *p*_*F*_).

Consider any PCI obtained by averaging: $\hat{p}=(p_{1}+p_{2}+\ldots +p_{N})/N$ (e.g., the (Overall) PCI is an average of Species PCIs; the Species Average Unanimous PCI is an average of Sample Unanimous PCIs; etc.). If the PCIs *p*_1_, *p*_2_,…, *p*_*N*_ are uncorrelated, the unbiased sample variance

$${\hat{\sigma }}^{2}=\frac{{\left(p_{1}-\hat{p}\right)}^{2}+{\left(p_{2}-\hat{p}\right)}^{2}+\ldots +{\left(p_{N}-\hat{p}\right)}^{2}}{N-1},$$ (6)

and the standard error mean $\hat{\sigma }/\sqrt{N}$ estimates the error in $\hat{p}$. Thus, the error in the (Overall) PCIs (*p*_*B*_, *p*_*U*_, *p*_*A*_, *p*_*F*_) can be estimated from the corresponding Species PCIs (*p*_*B*,*s*_, *p*_*U*,*s*_, *p*_*A*,*s*_, *p*_*F*,*s*_).

### Multiple testing

All p-values are given without multiple-test correction. As only unpublished preliminary datasets had uncorrected p-values p ≤ 0.05, the planned (Bonferroni) multiple-test correction for our published datasets is irrelevant.

### Supplementary parsimony and Bayesian analyses

To evaluate whether the species were recovered as monophyletic groups, the sequence alignment of the PCI dataset was analyzed under maximum parsimony (MP) using the heuristic search option in PAUPver4.0a [58], with a default setting for stopping the analysis. Phylogenetic trees were rooted with two sequences under *Gloeocantharellus persicinus* and *Gloeocantharellus purpurascens*. Gaps were treated as missing data [13]. Branch lengths equal to zero were collapsed to polytomies. Nonparametric bootstrap support [59] for each clades was assessed with the fast-step option, using 10,000 replicates, yielding a composite consistency index [60], retention index [61], and homoplasy index [61]. PAUP*Version 4.a selected as the best model for each partition ITS1, 5.8S, and ITS2 of sequences, as well as for their union ITS1+5.8+ITS2, so we had MrBayes 3.2 [62] do a Bayesian analysis [63, 64] with the model HSK+I+G. Two independent and simultaneous analyses starting from different random trees were run for two million generations with 12 parallel chains, and trees model scores saved every 100^th^ generation. The initial 1000 trees were discarded as burn-in before calculating the 50% majority-rule consensus tree and the posterior probability (PP) of the nodes, as described elsewhere [65]. A combination of bootstrap proportions and posterior probabilities was used to assess the level of confidence for a specific node [66]. The phylogenetic trees were viewed with FigTree v. 1.3.1 (http://tree.bio.ed.ac.uk/sotware/figtree/) and edited with Adobe Illustrator CS3 v.11.0.2 (Adobe Systems).

Perl Programs for calculating the PCIs are publicly available as open source software at https://go.usa.gov/xyuut.

## Supporting information

S1 Fig The 50% majority rule Bayesian tree inferred from ITS nrDNA assuming HSK + I + G model of Gomphales included in the PCI analyses. Terminal branches with names according to S1 Table. Colors: Blue, unique species names; Green, one species name and related infraspecific taxa; Red, clades including two or more species names; Orange: species names that occur in two or more clades. See S1 Table and S1 Fig for clades and singleton names.
(PDF)

S1 Text Morphological analyses and sequencing. (DOCX)

S2 Text Supplementary information about the barcoding analysis. (DOCX)

S1 Table Shows the PCI dataset, used both in PCI and phylogenetic analyses. The column headings are mostly self-explanatory. For human readability, some cells in some columns are highlighted: (A) Column “From authors” highlights sequences contributed by the authors; (B) Column “Typus from GenBank” highlights sequences declared as species types in GenBank; (C) Column “Length (nt)” highlights sequences of length less than 500 nt, our Short dataset; (D) Column “Ambiguous (nt)” highlights sequences with 10 or more ambiguous nucleotides; (E) Column “SH Number” highlights each missing UNITE identifier not found by entry of the GenBank accession number; and (F) Column “BLAST found SH” highlights which UNITE identifiers required the manual use of BLAST to find them from the GenBank accession number.
(XLSX)

S2 Table Shows all samples in the Total dataset not already presented in the PCI dataset. The Total dataset was used solely in the phylogenetic analysis. Column “Length (nt)” highlights sequences of length less than 500 nt, our Short dataset; and (D) Column “Ambiguous (nt)” highlights sequences with 10 or more ambiguous nucleotides.
(XLSX)
