# Correction: Figures of merit and statistics for detecting faulty species identification with DNA barcodes: A case study in *Ramaria* and related fungal genera

**DOI:** 10.1371/journal.pone.0250030

**Published:** 2021-04-07

**Authors:** María P. Martín, Pablo P. Daniëls, David Erickson, John L. Spouge

The Funding statement is incorrect. The correct Funding statement is as follows: “This study was funded by the U.S. National Library of Medicine (JS), Flora Micológica Ibérica PB95-0129-C03-01 and PB98-0538-C04-01 (PD), and Plan Nacional I+D+I CGL-2015-67459-P (MM.)”
