# Supplementary figures and images for: Figures of merit and statistics for detecting faulty species identification with DNA barcodes: A case study in Ramaria and related fungal genera
Source: PLoS One. 2020 Aug 19;15(8):e0237507. doi: 10.1371/journal.pone.0237507 (PMC7437900; doi:10.1371/journal.pone.0237507)

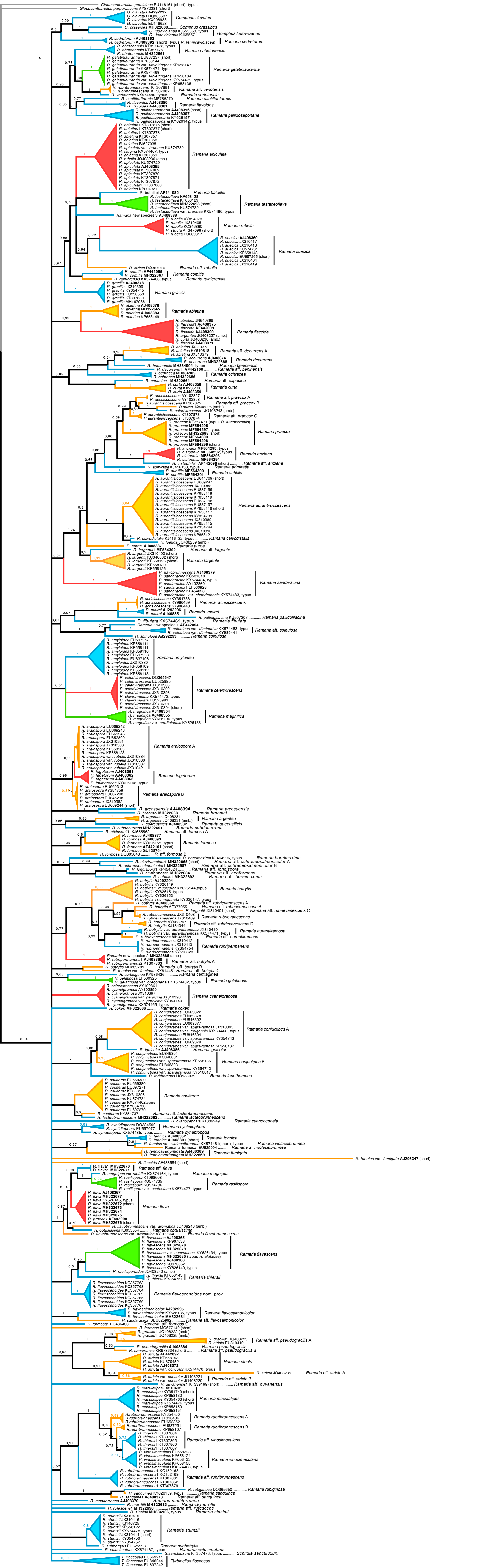

Supplement: S1 Fig — Terminal branches with names according to S1 Table. Colors: Blue, unique species names; Green, one species name and related infraspecific taxa; Red, clades including two or more species names; Orange: species names that occur in two or more clades. See S1 Table and S1 Fig for clades and singleton names. (PDF) [file pone.0237507.s001.pdf]
