# Supplementary material for: Figures of merit and statistics for detecting faulty species identification with DNA barcodes: A case study in Ramaria and related fungal genera
Source: PLoS One. 2020 Aug 19;15(8):e0237507. doi: 10.1371/journal.pone.0237507 (PMC7437900; doi:10.1371/journal.pone.0237507)
Supplement: S1 Text — (DOCX) [file pone.0237507.s002.docx]

**SI Appendix, Text 1. Morphological analyses and sequencing**

*Ramaria* is a worldwide genus of macromycetes with a tomentose base and branched basidiomata. The branches vary in both shape and color, and they often carry a smooth hymenophore. Because of the sporeprint, the hymenophore has a yellowish orange shade when mature, although usually in dried herbarium specimens the basidiome color fades to orange. The microscopy of *Ramaria* requires painstaking precision, and several factors complicate species identification, leading to contradictory expert opinions in a sparse taxonomic bibliography [1-6]. Morphologically, based on macroscopic characters a single species in *Ramaria* can vary widely in morphological appearance; and microscopically, its spores may be very variable in shape, and often ornamented with warts, grooves, crests or spines [7]. Sometimes, pictures or notes on fresh color and subsequent ephemeral color changes provide important characteristics for identifying species, so objective criteria using standardized color charts become useful. Experts on *Ramaria* have not even agreed on a standard color chart (e.g., see the color charts in [8-12]), however, and equivalence tables for translating between charts are usually lacking. To complicate the taxonomic task, moreover, the Index Fungorum (<http://www.indexfungorum.org/names/Names.asp>, accessed 25 September 2019) contains more than 600 names in the genus *Ramaria*, although some of these names actually belong to other genera such as *Clavaria*, *Clavulina*, *Clavulinopsis*, *Lentaria*, *Ramariopsis* or *Thelephora*. About 300 species probably belong to *Ramaria* *sensu stricto* [13]; but the Index Fungorum actually places hundreds of other taxa in *Ramaria* since there are many synonyms and invalid/illegitimate names.

Consequently, based only on macroscopic and microscopical characters, many European/American counterparts are difficult to separate (e.g.: *Ramaria fagetorum*/*R. subbotrytis*, *R. ignicolor*/*R. conjunctipes*, *R. flava*/*R. obtusissima*, *R. brunneicontusa*/*R. largentii*, *R. pallidosaponaria*/*R. flavigelatinosa*, *R. sanguinea*/*R. rubiginosa* or *R. flavosalmonicolor*/*R. sandaracina*), and European mycologists have in fact misidentified some [13-18]. Other species names common to both continents might also be misidentifications (e.g., *Ramaria subtilis*, *R. formosa*, *R. rubripermanens* or *R. botrytis*). Identification of some closely related species remains difficult (e.g., *Ramaria flavoides*/*R. abetonensis*), and some known species are probably species complexes requiring careful analysis [5, 19] (e.g., the *Ramaria ochraceovirens*/*R. ochrochlora*, *R. fennica*, *R. stricta*, *R. flava* or *R. botrytis* complexes).

Moreover, according to published molecular analyses [13, 20-26], some genera such as *Schildia*, *Gautieria*, *Phaeoclavulina*, *Turbinellus* or *Gomphus* are closely related to *Ramaria*. The independence of the genus *Phaeoclavulina* from *Ramaria* has been questioned [6, 16]; and the genus *Schildia*, likewise.

Thus, species delimitation is particularly challenging in the *Ramaria* genus and its allies.

**Morphological analyses**

The background on the taxonomy of *Ramaria* has been already studied mainly in last century [5, 18, 27-43] . Detailed notes on *Ramaria* taxonomy can also be found in Daniëls [6, 8, 13] from which we include the following notes:

**Macroscopic and organoleptic characteristics**

*Base and stipe —* In *Ramaria*, the basal tomentum continues into the substrate as mycelium and its micromorphic features are similar. The mycelium usually forms mycelial strands but in mycorrhizal *Ramaria* this rarely occurs. The morphology of the base provides an interesting source of data for the identification of some taxa.

*Colour* *—* Colour is one of the most used characteristics for species identification in *Ramaria* although it is also one of the most ephemeral. After drying or when aging due to the spore print on it, the basidioma acquires brownish, orange and yellowish shades independent of those of the fresh basidioma. For many species, the basidioma can change colour when bruised or cut. Branch apices may have different colours than the rest of the branches, especially when young. Thus, colour characterisation requires that all growth stages of the specimens be represented. The colour references should be taken from a standardised colour chart.

*Ramifications and branches* *—* Ramifications in mycorrhizal *Ramaria* are mainly polychotomous and umbelliform during the first stages of growth, giving to the basidiomata a cauliflower-like appearance. In saprobic *Ramaria* the ramifications are usually arborescent. There are basically two types of divisions: U-shaped when the splitting point, called valley or axil, is wide and rounded and V-shaped when it has a sharp angle. Ramification range is the number of splitting points of a branch from the base to the apex.

*Context* *—* The context consistency is a character frequently used for the discrimination of taxa, although data referring to context needs to be recorded while the specimens are still in a fresh condition. We can basically distinguish three types of consistencies: gelatinous, fibrous and coriaceous.

*Macrochemical reactions* *—* Many macrochemical tests are used in the genus, but we have only used iron salts, Melzer reagent and potassium hydroxide.

**Microscopic characteristics**

*Hyphal system and types of hyphae* *—* Two hyphal systems are variously represented in *Ramaria*: Monomitic and dimitic system, the latter formed by both generative and skeletal hyphae.

In generative hyphae, the most useful taxonomic character is the presence or absence of clamp connections, especially at the base of the basidia. Septa are sometimes inflated (ampulliform). The presence or absence of granular inward ornamentation on these vesicles is of taxonomic interest. The wall of generative hyphae is usually smooth, but in the mycelium and mycelial strands there are some hyphae partially covered by mucilaginous drops or some hyphae with granular walls or with short projections resembling acanthohyphae.

Skeletal hyphae exist in mycelial strands of some species of *Ramaria* subgen. *Lentoramaria*.

In some species, secretory hyphae arise from a septum, have a brownish to yellowish refringent content and sometimes have a terminal vesicle. In the trama, they are straight or linear but in the basal tomentum, mycelium or mycelial strands, they are often dendroid or with numerous short projections.

*Crystals from mycelium and mycelial strands* *—* Location and shape of the crystals have taxonomic value [13, 37]; at least six different types of crystals are found in Gomphales: tetragonal bipyramidal, star-shaped, acicular, rosette, amorphous and polygonal to cubic.

*Cystidia* *—* Some species of *Ramaria* subgen. *Echinoramaria* have star-shaped cystidia in the mycelial strands.

*Basidia* *—* There are mainly tetrasporic, with few species with bisporic basidia. The measurements of basidia were taken as follows: length is the distance from the basal septum to the basidial apex, excluding the sterigmata; width is the distance of the widest part of the upper third of the basidium. The most significant taxonomic character of the basidia is the presence or absence of a basal clamp.

Spores *—* The shape, size and ornamentation of the spore wall represent the best source of information at the microscopic scale. Size is characterised by three specific measurements: length (L), width (W) and length/width ratio (E): The values include length x width, mean length (Lm), mean width (Wm), and the mean length/width ratio (Em). Profile spore measurements include neither the apiculus nor ornamentation; the measurements were taken on material mounted in a 3% KOH solution with a minimum amount (n) of 20 spores per sample. Extreme values were not taken into consideration and measurement ranges were obtained by mean value ± standard deviation (σ). Although some *Ramaria* species have smooth spores, most species bear ornamented spores, with spinose, verrucose or striate ornamentation.

**DNA isolation, PCR and sequencing**

After morphological studies, the ITS nrDNA sequences were obtained from basidiomata as described in Telleria et al. [39]. BLAST+ 2.8.1 searches [40, 41], using MEGABLAST option were done to compare the sequences obtained against the sequences in the EMBL/GenBank/DDBJ databases [42, 43].

**References**

1. Corner EJH. The clavarioid Ramaria subgen. Echinoramaria. Persoonia. 1983;12:21–8.

2. Petersen RH. Response to E.J.H.Corner. Austral J Bot 1986;35:105–6.

3. Franchi P, Marchetti M. Una nova specie e una nuova combinazione nel genera Ramaria. Riv Micol 2017;60:299–302.

4. Christan J, Hahn C. Zur Systematik der Gattung Ramaria (Basidiomycota, Gomphales) Z Mykol 2005;71:7–42.

5. Christan J. Die Gattung Ramaria in Deutschland. Eching IHW Verlag; 2008. 352 p.

6. Daniels PP, Ribes Ripoll MA, Christan J. Notes on Gomphales V. Ramaria cokeri RH Petersen, first records for Macaronesia and Africa. Cryptogamie Mycologie. 2012;33(4):481-8. PubMed PMID: WOS:000314618800008.

7. Daniëls PP. Notas en Gomphales VII. Un abordaje taxonómico en Ramaria. Bol Soc Micol Extremeña 2016;20:48–53.

8. Séguy E. Code universal des couleurs. Paris: P. Lechevalier; 1936.

9. Kornerup A, Wanscher JH. Taschenatlas der Farben. Göttingen1981.

10. Ridgway R. Color standards and color nomenclature. Washington1912.

11. Küppers H. DuMont's Farbenatlas. 8 ed. Köln1999.

12. ISCC-NBS. Centroid Color Charts, U.S. Department of Commerce. Washington D.C.: National Bureau of Standards; 1964.

13. Daniëls PP. Estudio biosistemático del Orden Gomphales (Basidiomycota, Fungi) en la Peninsula Ibérica. Madrid: Univ. Complutense; 2002.

14. Bendiksen E, Bendiksen K, Brandrud TE, Kytövuori I, Toivonen M. Ektomykorrhizadannende korallsopper (Ramaria p.p.) i rik barskog i Norge. Agarica 2013;32:5–20.

15. Brandrud TE, Bendiksen E, Bendiksen K, Kytövuori I, Toivonen M. Ectomycorrhizal Ramaria species in nutrientpoor Fennoscandian conifer forests including a note on the Ramaria botrytis complex. Agarica. 2012;36:89–108.

16. Christan J, Mata M. Ramaria isaaci sp. nov. and Ramaria gracilispora sp. nov. two new Ramaria from Costa Rica, with notes on Genus Phaeoclavulina Brinkmann (1897) ss. Giachini 2011. Mycol Bav 2012;13:45–57.

17. Schild E. Ramaria-Studien. Z Mykol 1982;48:117–28.

18. Franchi P, Marchetti M. Fungi non delineati. Pars XVI. Introduzione allo studio del genere Ramaria in Europe. Alassio, Italy: Edt. Candusso; 2001. 104 p.

19. Bendiksen K, Kytovuori I, Toivonen M, Bendiksen E, Brandrud TE. Ectomycorrhizal Ramaria species in nutrientpoor Fennoscandian conifer forests including a note on the Ramaria botrytis complex. Agarica 2015;36:89–108.

20. Hosaka K, Bates ST, Beever RE, Castellano MA, Colgan W, Dominguez LS, et al. Molecular phylogenetics of the gomphoid-phalloid fungi with an establishment of the new subclass Phallomycetidae and two new orders. Mycologia. 2006;98(6):949-59. PubMed PMID: WOS:000245858800012.

21. Humpert AJ, Muench EL, Giachini AJ, Castellano MA, Spatafora JW. Molecular phylogenetics of Ramaria (Gomphales) and related genera: evidence from nuclear large subunit and mitochondrial small subunit rDNA sequences. Mycologia 2001;93:465–77. doi: 10.2307/3761733.

22. Ando Y, Christan J, Maekawa N, editors. Polyphyly of Phaeoclavulina (Ramaria subgenus Echinoramaria) revealed from mitochondrial and nuclear sequence data. International Mycological Congress, IMC9; 2010 1-6 August, 2010; Edinburgh, U.K.

23. Giachini AJ, Hosaka K, Nouhra E, Spatafora J, Trappe JM. Phylogenetic relationships of the Gomphales based on nuc-25S-rDNA, mit-12S-rDNA, and mit-atp6-DNA combined sequences. Fungal Biol 2010;114:224–34. doi: 10.1016/j.funbio.2010.01.002.

24. Petersen RH, Hughes KW, Justice J. Two new species of Ramaria from Arkansas. MycoKeys 2014;8:17–29. doi: 10.3897/mycokeys.8.7356.

25. Franchi P, Marchetti M. Schildia, un nuovo Genere delle Gomphales e due nuove Ramaria della Toscana. Funghi clavarioidi - VIII. Riv Micol 2015;58:99–130.

26. Das K, Hembrom ME, Dutta AK, Parihar A, Paloi S, Acharya K. Ramaria subalpina (Gomphaceae): a new edible fungus from India. Phytotaxa. 2016;246(2):137-44. PubMed PMID: WOS:000370223600005.

27. Coker WC. The Clavariaceae of the United States and Canada: University North Carolina Press; 1923.

28. McAfee BJ, Grund DW. The clavarioid fungi of Nova Scotia. Proc Nova Scotian Inst Sci 1981;32:1-73.

29. Schild E. Die Sektion Flaccidae der Gattung Ramaria. Schweiz Z Pilzk 1978;108:97-102.

30. Schild E. Studie über Ramarien. Z Pilzk 1981;122:169–78.

31. Schild E. Studie über Ramarien II Mycol Helv 1983;1(1):47-57.

32. Schild E. Ramaria – Studien. Z Mykol 1983;56(1):131-50.

33. Schild E. Eine Analyse des Ramaria – fennica – fumigata – versatilis – Komplexes. Z Mykol 1995;61(2):139-82.

34. Schild E. Il genere Ramaria: Cinque nuovi taxa dall’ Italia mediterranea. Riv Micol. 1998;41(2):119-40.

35. Schild E. Die Gattung Ramaria: 4 neue Arten aus Italien und Sardinien. Z Mykol. 1998;64(1):53–66.

36. Schild E. Vier neue aus Ramaria-Arten der Schweiz, Italien und Slowenien Z Mykol. 2003;69(1):101–22.

37. Christan J. Bestimmungsmerkmale im Basismycel. Beispiele aus der Gattung Ramaria. Z Mykol 2003;69(2):299-305.

38. Marr CD, Stuntz DE. Ramaria of Western Washington. Bibl Mycol 1973;38:1–232.

39. Telleria MT, Dueñas M, Martín MP. Three new species of Hydnophlebia (Polyporales, Basidiomycota) from Macaronesian Islands. MycoKeys. 2017;27:39–64. doi: 10.3897/mycokeys.27.14866.

40. Altschul SF, Gish W, Miller W, Myers EW, Lipman DJ. Basic Local Alignment Search Tool. Journal of Molecular Biology. 1990;215(3):403-10. doi: 10.1016/S0022-2836(05)80360-2.

41. Altschul SF, Madden TL, Schaffer AA, Zhang J, Zhang Z, Miller W, et al. Gapped BLAST and PSI-BLAST: a new generation of protein database search programs. Nucleic Acids Res. 1997;25(17):3389-402.

42. Cochrane G, Karsch-Mizrachi I, Nakamura Y. The International Nucleotide Sequence Database Collaboration. Nucleic Acids Research. 2011;39:D15-D8. doi: 10.1093/nar/gkq1150. PubMed PMID: WOS:000285831700004.

43. Cochrane G, Karsch-Mizrachi I, Takagi T. The International Nucleotide Sequence Database Collaboration. Nucleic Acids Research. 2016;44(D1):D48-D50. doi: 0.1093/nar/gkv1323. PubMed PMID: WOS:000371261700006.
