# Supplementary material for: Figures of merit and statistics for detecting faulty species identification with DNA barcodes: A case study in Ramaria and related fungal genera
Source: PLoS One. 2020 Aug 19;15(8):e0237507. doi: 10.1371/journal.pone.0237507 (PMC7437900; doi:10.1371/journal.pone.0237507)
Supplement: S2 Text — (DOCX) [file pone.0237507.s003.docx]

**SI Appendix, Text 2. Supplementary Information about the Barcoding Analysis**

**Sequence analyses**. In our PCI dataset, sequence lengths ranged from 207 nt to 785 nt, and the average length was 582.8 nt with standard deviation 75.3 nt. The unique sequences contained 222,621 nt, with base composition A (20.80%), C (26.07%), G (25.90%), T (26.93%), with 0.30% bases ambiguous. The Muscle3.8.31 multiple sequence alignment of unique sequences had 1478 columns and inserted 341,975 gap characters, so gaps constituted 60.57% of the positions.

In our Long dataset, sequence lengths ranged from 501 nt to 785 nt, and the average length was 601.4 nt with standard deviation 42.28 nt. The unique sequences contained 208,667 nt with base composition A (20.75%), C (26.10%), G (26.93%), T (26.91%), with 0.31% bases ambiguous. The Muscle3.8.31 multiple sequence alignment of unique sequences had 1439 columns and inserted 290,666 gap characters, so gaps constituted 58.21% of the positions.

In our Taxonomic dataset, sequence lengths ranged from 207 nt to 785 nt, and the average length was 582.5 nt with standard deviation 78.8 nt. The unique sequences contained 87,951 nt with base composition A (20.73%), C (25.89%), G (26.23%), T (26.00%), with 0.14% bases ambiguous. The Muscle3.8.31 multiple sequence alignment of unique sequences had 1,204 columns and inserted 93,853 gap characters, so gaps constituted 51.62% of the positions.

**A Taxonomic Unanimity Test.**

Fig. S2 illustrates a 2x2 table relevant to a species *s* with a total of *N* = *A*+*B*+*C*+*D* samples (*N* more than 2). Each sample within the species had a source of taxonomic identification, either expert or not. Of the *N* samples, *A*+*C* had expert species identification, whereas *B*+*D* had not. Similarly, each sample within species *s* was either unanimous (had nearest neighbors all belonging to species *s*) or not. In Fig. S2, of the *N* samples, *A*+*B* were unanimous, whereas *C*+*D* were not.


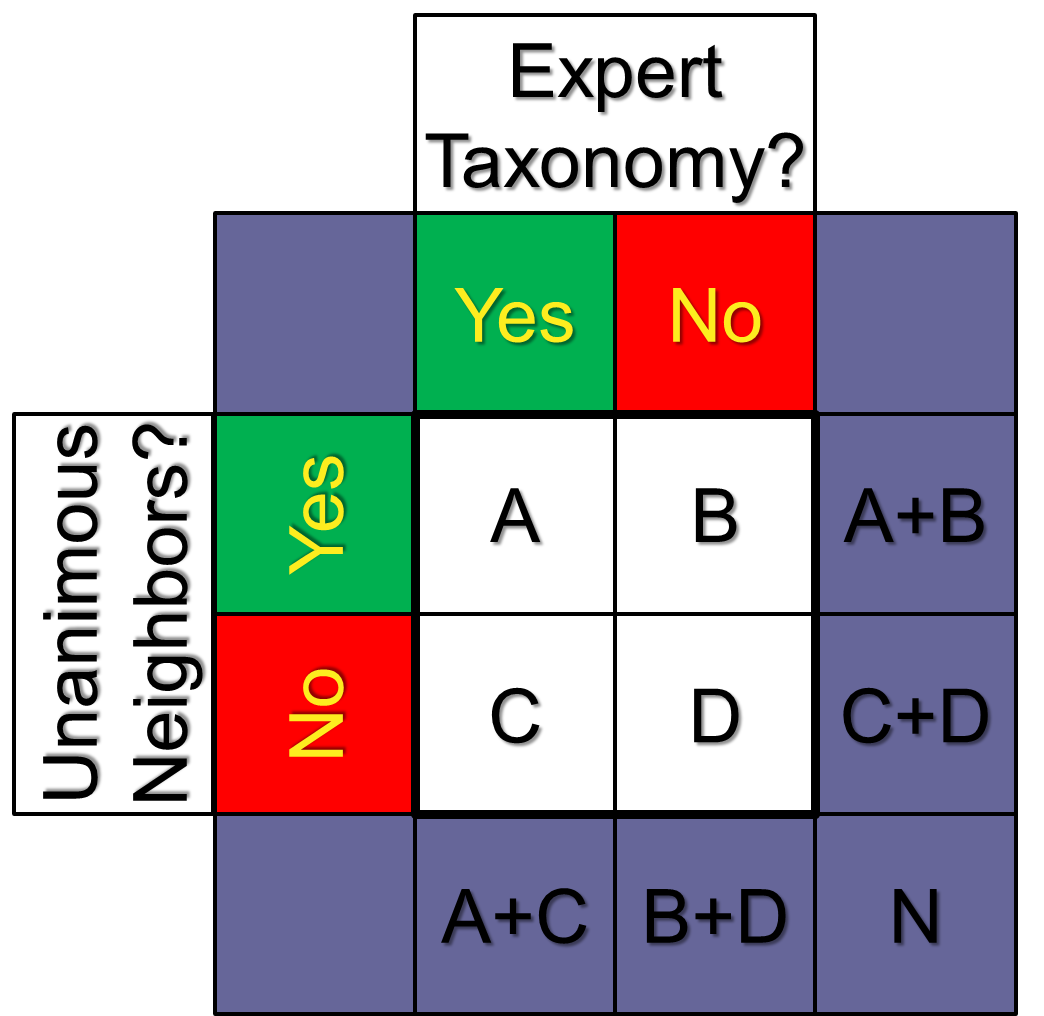


**Fig.** S2**.** An illustrative 2x2 table for a single species, showing counts *A*, *B*, *C*, and *D*. It also shows marginal counts and the total number of samples, *N* = *A*+*B*+*C*+*D*.

The taxonomic unanimity test in the Theory section of the main article yields a z-score *Z* having the same formal expression as the log-rank statistic used to analyze the difference between two Kaplan-Meier survival curves. Under a proportional hazards model [[1, p.91](#_ENREF_1)], the log-rank statistic permits statisticians to infer that the treated and control patient groups have differing hazard rates for death [[2](#_ENREF_2)]. Conceptually, the log-rank statistic analyzes a series of 2x2 tables, one for each time-point, where treated and control patients either survive or die between the time-points. In the taxonomic setting, each 2x2 table corresponds to a species instead of a time-point. Conceptually, the taxonomic unanimity test loses the temporal relationships among the 2x2 tables of the log-rank test, but retains the formal mathematical manipulations to produce a z-score with an approximately Gaussian distribution.

**Tables for detecting misannotated samples**.

Table S3. Species with possible misannotations in the GenBank dataset.

| Species Binomial | Sample Identifier of GenBank Sequences |
| --- | --- |
| *Ramaria abietina* | FJ627035  JN649369  KP004921  KT307858  KT307859 |
| *Ramaria botrytis* | AF377055  KY626149  KY626153  MH289789 |
| *Ramaria coulterae* | KY354737 |
| *Ramaria curta* | JQ408230 |
| *Ramaria fennicavarfumigata* | KX814451 |
| *Ramaria flaccida* | AF438554 |
| *Ramaria flavescens* | KP967538 |
| *Ramaria formosa* | EU525994 |
| *Ramaria rubrievanescens* | EU669317 |
| *Ramaria sandaracina* | EU525992  KP454028 |
| *Ramaria stricta* | DQ367910  EU819419  JQ408235  AF347098 |
| *Ramaria strictavarconcolor* |  |
| *Ramaria stuntzii* | JX310414 |

Table S3 contains samples in the GENBANK DATASET that had at least one nearest neighbor (under p-distance) from a different species. If a species *s* does not appear in Table S3, either every sample or no sample from it: (1) was in the TAXONOMIC DATASET; OR (2) had all its nearest neighbors belonging to *s*. (Table S3 lists the species *Ramaria strictavarconcolor* without GenBank samples, because no GenBank sample from *Ramaria strictavarconcolor* had a nearest neighbor outside of *Ramaria strictavarconcolor*). If a species does not appear in Table S3, therefore, its results do not discriminate between the accuracy of species annotations in the GENBANK and TAXONOMIC DATASETs. Table S3 contains all samples where barcoding analysis might suggest having an expert check a GenBank annotation.

Table S4. Species with possible barcoding misidentifications because the ITS sequence was less than 500 bp.

| Species Binomial | Sample Identifier of Short Sequences |
| --- | --- |
| *Ramaria abietina1* |  |
| *Ramaria araiospora* |  |
| *Ramaria cedretorum* | AJ408392 |
| *Ramaria celerivirescens* |  |
| *Ramaria fennicavarfumigata* | AJ296347 |
| *Ramaria flaccida* | AF438554 |
| *Ramaria flava* | MH322672 |
| *Ramaria formosa* |  |
| *Ramaria largentii* | JX310401 |
| *Ramaria praecox* |  |
| *Ramaria stricta* | AF347098 |
| *Ramaria stuntzii* | JX310414 |

Analogously to Table S3, Table S4 contains samples in the SHORT DATASET that had at least one nearest neighbor (under p-distance) from a different species. If a species *s* does not appear in Table S4, either every sample or no sample from it: (1) was in the LONG DATASET; OR (2) had all its nearest neighbors belonging to *s*. If a species does not appear in Table S3, therefore, its results do not discriminate between the accuracy of species annotations in the SHORT and LONG DATASETs. Table S4 contains all samples with short sequences where the barcoding analysis might suggest the sequence length might have caused species misidentification.

**A Taxonomic Signed-rank Test of Improvements to a Barcoding Pipeline.** Consider a pipeline *A*, which provides a current standard for barcode species identification. Another pipeline *B* (possibly varying a single step in pipeline *A*) may improve on pipeline *A*. A natural question arises: how can one demonstrate the improvement statistically? A one-sided Wilcoxon matched-pair signed-rank test [[3, p. 75-83](#_ENREF_3)] can decide whether pipeline *B* really improves on pipeline *A*, as follows.

Fix any of the Species PCIs, e.g., the Species Fractional PCI $p_{F,s}$. Each pipeline produces a PCI $p_{F,s}$ for each species *s*, denoted $p_{F,s}^{\left(A\right)}$ or $p_{F,s}^{\left(B\right)}$. Ignore species *s* if $p_{F,s}^{\left(A\right)}=p_{F,s}^{\left(B\right)}$ are tied. Rank the absolute differences $\left|p_{F,s}^{\left(B\right)}-p_{F,s}^{\left(A\right)}\right|$ of the PCI pairs of the remaining $n$ species; and add a minus sign to the ranks if $p_{F,s}^{\left(A\right)}-p_{F,s}^{\left(B\right)}>0$. Under a null hypothesis $H_{0}$ that the two pipelines produce a distribution of PCI differences symmetric around 0, and that the differences are probabilistically independent, the Wilcoxon matched-pair signed-rank test applies.

Table S5. Signed-rank test comparison of different pipelines on the TAXONOMIC DATASET

| PCI | Align & Extract  Pipeline *A&E* | Extract & Align  Pipeline *E&A* | Signed-rank Test | | |
| --- | --- | --- | --- | --- | --- |
|  |  |  | *n* | p-value |  |
| Fractional ($p_{F}$) | $0.863\pm0.063$ | $0.886\pm0.049$ | 7 | 0.952 |  |
| Average Unanimous ($p_{A}$) | $0.846\pm0.067$ | $0.886\pm0.049$ | 7 | 0.714 |  |
| Unanimous ($p_{U}$) | $0.654\pm0.093$ | $0.769\pm0.083$ | 5 | 1.000 |  |
| Barcode Gap ($p_{B}$) | $0.577\pm0.097$ | $0.692\pm0.091$ | 3 | 0.125 |  |

The results for the signed-rank test on Taxonomic dataset are in the same format as Table 1.

**Resampling the PCI dataset.** Consider a barcode dataset of $N$ samples. To examine the robustness of a PCI to sparser sampling or other random changes in the dataset, resample $n$ samples (without replacement, uniformly) at random from the $N$ samples in the dataset to mimic the collection of a smaller sample ($n<N$), and then calculate the PCI of interest. Repeat the procedure many times (here, 1000 times) to determine the mean and sample standard error of the resampled PCI. The spirit of the approach is similar to the statistical bootstrap [[4](#_ENREF_4)]: as the dataset size $N$ approaches the population size, if $n$ is much smaller than $N$, then random dataset mimics the collection of a dataset of $n$ samples.

Although the quality of multiple sequence alignment can affect the accuracy of species identification, according to the Wilcoxon ranked-sign test in Tables 2 and S4, realigning subsets of the PCI dataset (Pipeline *E&A*) did not significantly improve PCIs over reusing the sequence alignment from the PCI dataset (Pipeline *A&E*). Our resampled datasets therefore reused the sequence alignment from the PCI dataset. The reused sequence alignment permitted precomputation and reuse of p-distances from the PCI dataset, making the speed of the resampling computations practical. As a secondary consideration, sequence alignment reuse also controls for any effect of sequence realignment on species identification.

## References

1. Cox DR, Oakes D. Analysis of Survival Data. London: Chapman and Hall; 1984.

2. Peto R, Peto J. Asymptotically efficient rank invariant test procedures. J R Stat Soc Series A-General. 1972;135:185-98. doi: 10.2307/2344317. PubMed PMID: WOS:A1972N166900020.

3. Siegel S. Nonparametric Statistics for the Behavioral Sciences. 1 ed. New York: MacGraw-Hill; 1956.

4. Efron B, Tibshirani RJ. An Introduction to the Bootstrap. New York: Chapman and Hall; 1993.
